# The impact of physicochemical features of carbon electrodes on the capacitive performance of supercapacitors: a machine learning approach

**DOI:** 10.1038/s41598-023-33524-1

**Published:** 2023-04-20

**Authors:** Sachit Mishra, Rajat Srivastava, Atta Muhammad, Amit Amit, Eliodoro Chiavazzo, Matteo Fasano, Pietro Asinari

**Affiliations:** 1 Department of Energy “Galileo Ferraris”, Politecnico di Torino, Corso Duca degli Abruzzi 24, 10129 Turin, Italy; 2 IMDEA Network Institute, Universidad Carlos III de Madrid, Avda del Mar Mediterraneo 22, 28918 Madrid, Spain; 3 Department of Engineering for Innovation, University of Salento, Piazza Tancredi 7, 73100 Lecce, Italy; 4 Department of Mechanical Engineering, Mehran University of Engineering and Technology, SZAB Campus, Khairpur Mir’s, Sindh 66020 Pakistan; 5 Istituto Nazionale di Ricerca Metrologica, Strada delle Cacce 91, 10135 Turin, Italy

**Keywords:** Energy storage, Materials for devices, Computational methods

## Abstract

Hybrid electric vehicles and portable electronic systems use supercapacitors for energy storage owing to their fast charging/discharging rates, long life cycle, and low maintenance. Specific capacitance is regarded as one of the most important performance-related characteristics of a supercapacitor’s electrode. In the current study, Machine Learning (ML) algorithms were used to determine the impact of various physicochemical properties of carbon-based materials on the capacitive performance of electric double-layer capacitors. Published experimental datasets from 147 references (4899 data entries) were extracted and then used to train and test the ML models, to determine the relative importance of electrode material features on specific capacitance. These features include current density, pore volume, pore size, presence of defects, potential window, specific surface area, oxygen, and nitrogen content of the carbon-based electrode material. Additionally, categorical variables as the testing method, electrolyte, and carbon structure of the electrodes are considered as well. Among five applied regression models, an extreme gradient boosting model was found to best correlate those features with the capacitive performance, highlighting that the specific surface area, the presence of nitrogen doping, and the potential window are the most significant descriptors for the specific capacitance. These findings are summarized in a modular and open-source application for estimating the capacitance of supercapacitors given, as only inputs, the features of their carbon-based electrodes, the electrolyte and testing method. In perspective, this work introduces a new wide dataset of carbon electrodes for supercapacitors extracted from the experimental literature, also giving an instance of how electrochemical technology can benefit from ML models.

## Introduction

**Figure 1:**
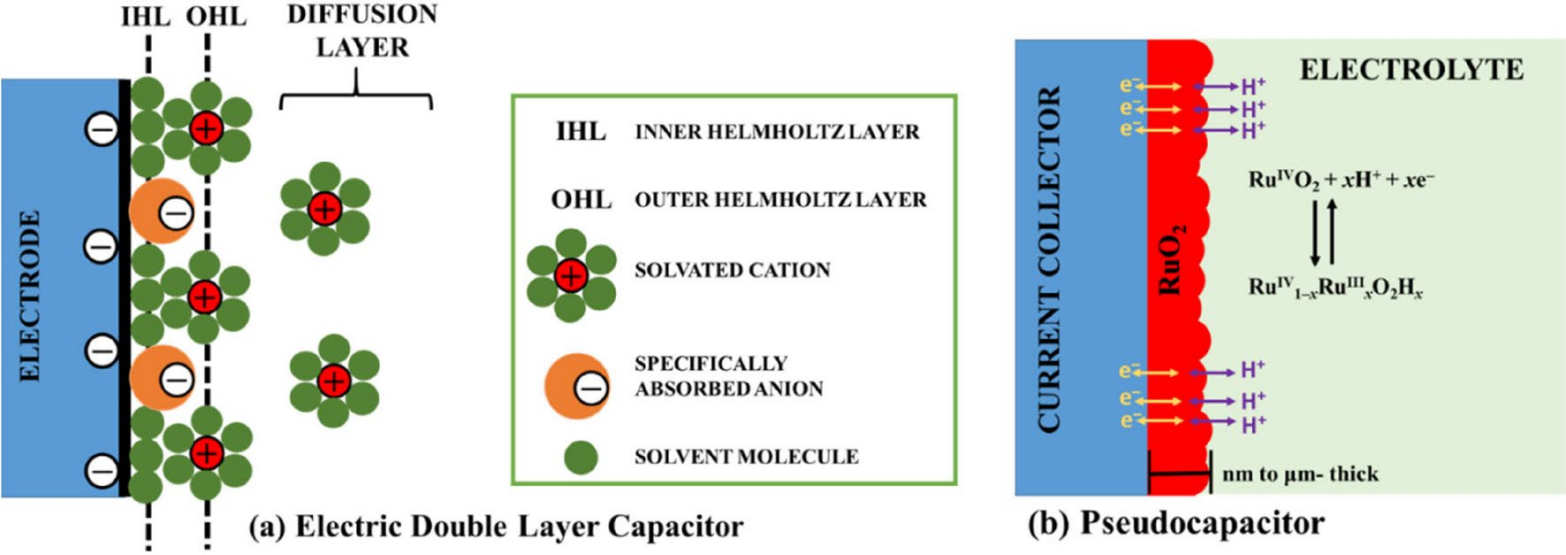
Schematic diagram of supercapacitors: (**a**) Electric double-layer capacitor (EDLC); (**b**) example of pseudocapacitor based on ruthenium oxide.

Electrochemical capacitors (supercapacitors) are electrochemical devices that are extensively used for energy storage due to promising characteristics such as high-power density, electrochemical stability, fast charge/discharge rates, safe operation mode, high power density, and long cycle life^1–3^. These characteristics enable their use in a broad range of energy storage applications, e.g., for hybrid electric vehicles, portable electronics, and memory backup systems^4–6^. Other than energy storage, there are some further interesting applications of supercapacitors such as heat-to-current conversion of low-grade thermal energy^7^ and renewable energy extraction using a supercapacitor from water solutions^8^. Supercapacitors have been primarily classified into two types based on their charge storage mechanism: (i) electric double-layer capacitors (EDLCs), which store electrical charge via ion adsorption at the electrode surface, and (ii) pseudo-capacitors, which store charges via reversible Faradaic redox reactions (see Fig. 1). Generally, EDLCs have superior cycle stability but lower specific capacitance in comparison to pseudo-capacitors, which have a high specific capacitance but a low power density and poor cycle stability instead^9,10^. The current study focuses on EDLC supercapacitors and their optimization.

For the optimization of the electrochemical performance of EDLC supercapacitors, it is critical that the electrode materials have commendable physicochemical properties, including appropriate pore size distribution, high specific surface area, high electrical conductivity, as well as electrochemical and mechanical stability for good cycling performance^9^. Numerous materials have been synthesised and used as supercapacitor electrodes in recent years, including porous carbon^11–13^, hierarchical porous carbon^14–16^, activated carbon^10,17,18^, graphene^9,19,20^, rGO-PANI nanocomposite^21^, carbon nanotubes^22–24^, In_2_O_3_-loaded porous carbon^25^, and carbon aerogels^26,27^. Among these electrode materials, carbon is the most frequently used due to its versatility and uniqueness^28^. It exists in various forms (e.g., graphite, diamond), dimensionalities (fibres, fabrics, foams, and composites), ordered and disordered structures (depending on the degree of graphitization), with commendable electrical conductivity^29,30^. The catalytic, optical, mechanical, and electrochemical properties of carbon make it an excellent material for energy conversion and storage applications^31^. Additionally, its well-established synthesis and activation methods enable its use as an electrode in supercapacitors with an appropriate pore size distribution^32^.

Porous and activated porous carbon (AC) and hierarchical porous carbon (HPC) have been proposed in the literature for carbon electrodes (see Supplementary Note 1 for a detailed review). Because of its high specific surface area (SSA), improved electrical conductivity, adjustable pore sizes, electrochemical stability and low cost, porous carbon offers significant promise for use as the electrode^33–36^. These properties make AC an excellent material for a variety of applications, including water purification, gas separation and storage, and electrode materials for capacitors, fuel cells and batteries^37^. Differently from AC, hierarchical porous carbon material contains pores in a wide range of length scales, namely macro- (> 50 nm), meso- (2–50 nm), and micro- (< 2 nm) scales. The presence of macropores in HPC allows high-rate ion transport and acts as an ion reservoir. Furthermore, the interconnected mesopores provide low resistance pathways for the diffusion of ions; whereas the high SSA of micropores enhances the adsorption of ions at the pore surface^38^. These unique properties of HPC gained recent interest in the selection of electrode materials for supercapacitors.

Besides the SSA and pore volume, there are also several other factors that influence performance of electrodes in supercapacitors, such as surface functional groups and conductivity. These can be modified by introducing heteroatoms—HA (nitrogen, oxygen, sulphur, etc.) in the carbon electrodes, which do not only enhance the wettability, but also improve electronic conductivity of activated carbon^39^. Nitrogen doping on carbonaceous material as electron donor is useful for enhancing the specific capacitance via faradaic reaction and enhancing wettability^16^. Similarly, oxygen doping improves the surface wettability, which in turn improves the supercapacitor performance^39^. Sulphur doping on carbonaceous material increases its bandgap, thus enhancing the electron donor properties and changing the electronic density of state. Sulphur doping also increases wettability, which in turn decreases the diffusion resistance that occurs between the electrode and electrolyte ions^40^.

In perspective, graphene is a promising electrode material for supercapacitors too, due to a high electrical conductivity, high SSA, and excellent mechanical strength^41,42^. Its porous structure also facilitates charge transport in the supercapacitor. The SSA of graphene is highly tuneable according to the requirement of supercapacitor electrode for energy storage applications. Also, the presence of highly movable free $\pi$ electrons on its orbital are responsible for the exceptionally high electrical conductivity^41^. Furthermore, the electrical behaviour of graphene can be improved through functionalization^43^ and heteroatom doping^44^.

Numerous attempts have been made to increase the specific capacitance of supercapacitors by utilizing different types of carbon electrodes with varied pore size distributions, high specific surface area, diverse morphologies, and modified surface chemistry^45^. However, the influence of these physicochemical parameters on the specific capacitance of supercapacitors has not been completely understood. Additionally, conventional theories and models are incapable of capturing with sufficient accuracy the microscopic details of the underlying physical mechanisms affecting ion transport, which are essential for accurately predicting the capacitive performance of supercapacitors. Recent advances in machine learning (ML) algorithms and their application to physics-based systems have made it possible to recognize the effects of various physicochemical features of carbon-based electrode materials in enhancing the specific capacitance of supercapacitors. In detail, Zhu et al.^46^ used artificial neural network (ANN) algorithm to predict the specific capacitance of carbon-based supercapacitors. They collected 681 data entries from the published experimental papers, with information about specific surface area, pore size, presence of defects, nitrogen doping level, and potential window. The authors concluded that ANN yields better predictability of specific capacitance than linear regression and Lasso methods. However, the ANN method could not discriminate the impact of each feature separately. Instead, Su et al*.*^47^ interpolated the specific capacitance of carbon-based electric double layer capacitors using four different ML models, namely linear regression (LR), support vector regression (SVR), multilayer perception (MLP), and regression tree (RT). The authors ranked the performance of the different ML models as follows: RT > MLP > SVR > LR. They found out that the specific surface area, potential window, and heteroatom doping enhance the specific capacitance of EDLC supercapacitors. Nevertheless, the authors did not analyse the effect of pore volume and size of electrodes. Finally, Zhou et al*.*^48^ proposed a ML model to determine the features with stronger impact on the specific capacitance and power density of supercapacitors, limiting their analysis only to activated carbon materials for the electrodes and a 6 M KOH electrolyte.

Although some data-driven analyses of the relation between a few features of supercapacitors and their specific capacitance have been reported in previous studies, a comprehensive study on more physicochemical features, electrode materials, methods of testing and electrolytes has been hindered by the limited number of entries in the considered database. In this work, we first created a larger dataset by extracting data from 147 experimental research articles on supercapacitors comprising carbon-based electrodes. The resulting curated dataset is made of 4899 entries and primarily contains information about the specific surface area, the presence of defects, the pore volume and size of pores, the potential window, the current density as well as the nitrogen and oxygen content of the carbon-based electrode materials. Additionally, the importance of categorical variable such as testing method, electrolyte, and carbon structure of the electrode on the specific capacitance was studied for the first time. ML algorithms were then applied to this dataset to identify those characteristics of the electrode material that significantly affect their capacitive performance, and to develop the best model possible for predicting the specific capacitance of supercapacitors. To ease the transferability of results, we developed SUPERCAPs, an open-source software to estimate the specific capacitance of carbon-based EDLC according to the structural features of electrodes, the electrolyte solution and method of testing.

## Materials and methods

### Dataset creation

To develop the dataset, we extracted information from 147 research articles on carbon-based electrode supercapacitors, collecting 4899 data entries (see the Supplementary Note 2 for a detailed list of data sources). Each data entry includes information related to carbon electrodes (i.e., pore size, pore volume, etc.), the test system (i.e., electrolyte, potential window, current densities), and the resulting specific capacitance. The latter is defined as $C = \frac{\varepsilon S}{d},$ where, *C*, *ε*, *S*, and *d*, are the specific capacitance, permittivity of electrolyte, surface area of electrode–electrolyte interface, and charge separation distance, respectively.

The various parameters included in the dataset that characterize the electrodes and the test system are as follows:

1. *Specific surface area *(*SSA, *[m^2^/g]) The specific capacitance of EDLC supercapacitors depends on the adsorption of electrolyte ions on the electrode surface and directly depends on the surface area of the electrode material. Thus, to enhance the specific capacitance, a high specific surface area of electrode material is preferable^1,29^.
2. *Pore size *(*PS, *[nm]) The presence of micro/mesopores in carbon-based electrodes provides efficient pathways for the electrolyte ions transports, which leads to rapid ionic diffusion in the supercapacitor^49–51^.
3. *Pore volume *(*PV *[cm^3^/g]) This feature is related to PS, with an additional normalization with respect to the mass of the electrode.
4. *Ratio between D and G peaks *(*I*_*D*_*/I*_*G*_*, [-]*) The high ratio of intensities between peaks D and G represents the increase in defects, which leads to a decrease in the electrical conductivity of carbon-based electrodes. The decrement in the electrical conductivity of electrode material affects the capacitive performance of the supercapacitor^49^.
5. *Nitrogen content in the electrode *(*N%, *[%]) The nitrogen doping in the carbon matrix electrode material improves the specific capacitance by Faradaic reaction. It does not only enhance the charge mobility on carbon surfaces, but it also increases its wettability^16,52^.
6. *Oxygen content in the electrode *(*O%, *[%]) The oxygen content in the electrode material improves the wettability of the electrode surface in the electrolyte, which enhances the electrochemical performance of the supercapacitor^39^.
7. *Sulphur content in the electrode *(*S%, *[%]) Sulphur is most reactive element among heteroatom doping elements due to its unpaired electrons and wider bandgap. Increased specific capacitance results from the sequence of Faradaic reactions on sulphur doped carbonaceous materials^40^.
8. *Potential window *(*PW, *[V]) Potential window is a range of potentials in which no Faradaic reaction occurs, implying that material and electrolyte are stable when the potential is applied in this range. It is dependent on the type of material and electrolyte.
9. *Current density *(*I, *[A/g]) In porous carbonaceous electrodes, ions of the electrolyte do not have sufficient time to reach the microporous surface of the electrode at a high current density due to a fast-charging rate. Therefore, increasing current density degrades the capacitive performance of the supercapacitor^53,54^.

Furthermore, the following categorical variables have been also included in the dataset:

1. *Electrolyte type* The type of electrolyte is crucial to supercapacitor performance. A good electrolyte has a broad potential window, strong electrochemical stability, high ionic concentration, and conductivity. Electrolytes are classified into three types: aqueous, organic liquid and ionic liquid^55^.
2. *Method of testing specific capacitance* Two-electrode and three-electrode method are the two methods for evaluating the specific capacitance. The two-electrode method consists of working and counter electrodes, where the potential is supplied, and the resultant current is obtained at either working or counter electrode. The three-electrode system consists of working electrode, counter electrode, and reference electrode. The reference electrode serves as a reference for measuring and adjusting the working electrode potential, without transmitting any current.
3. *Electrode structure* AC, HPC, and heteroatom (HA)-doped electrodes have a significant effect on their performance, as comprehensively discussed in the Supplementary Note 1.

**Figure 2:**
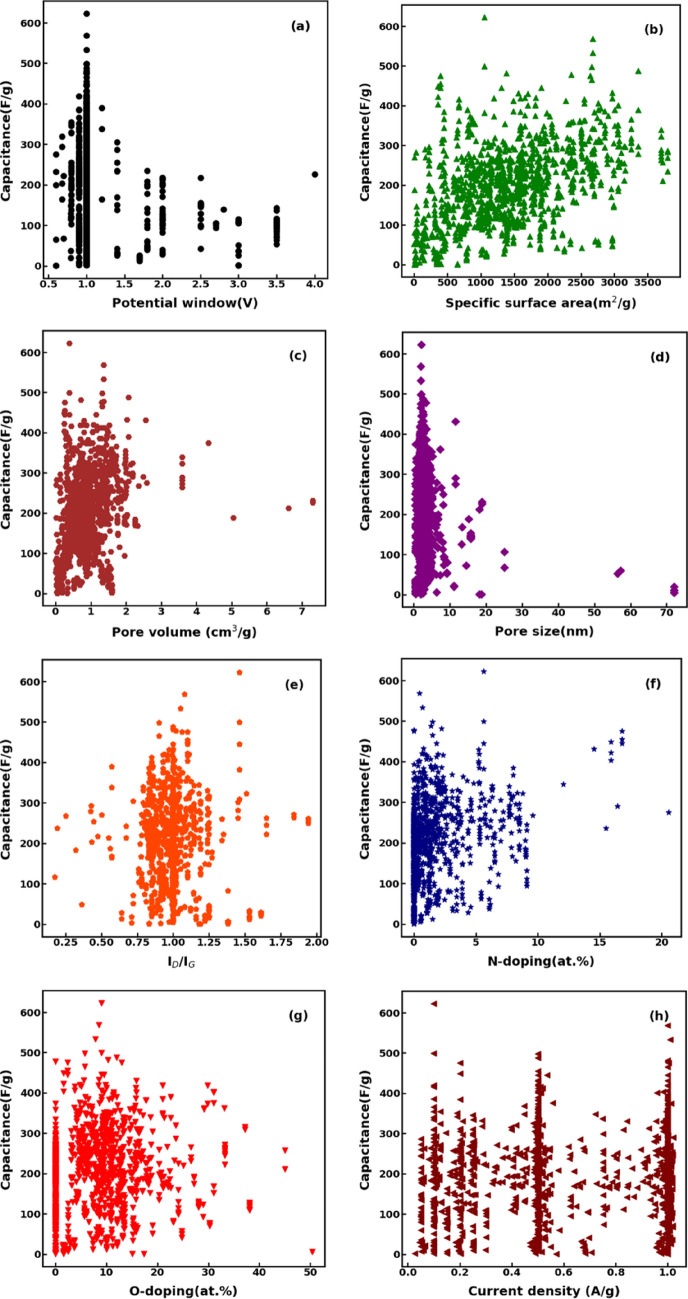
Relation between the specific capacitance of supercapacitors in the curated dataset and (**a**) potential window, (**b**) specific surface area, (**c**) pore volume, (**d**) pore size, (**e**) I_D_/I_G_, (**f**) N-doping (wt%), and (**g**) O-doping (wt%) at current density of I = 1 A/g. (h) Relationship between specific capacitance and different current densities.

Figure 2a–g shows the influence of the various physicochemical parameters on the specific capacitance of supercapacitors at a current density of 1 A/g for the whole dataset (note that, due to lack of data at 1 A/g, the relationship between specific capacitance and sulphur doping percentage is not presented); whereas Fig. 2h shows the relationship between specific capacitance and different current densities. While SSA shows a certain correlation with $C$ in agreement with previous results^1,29^, the other features of carbon-based supercapacitors have a less clear and nonlinear influence on the specific capacitance, therefore requiring advanced data analysis tools to fully understand it.

Prior to applying the regression algorithms to the dataset containing the physicochemical properties and specific capacitance of carbon-based electrodes, it is necessary to pre-process the data to remove possible gaps and outliers. The process of improving data quality is known as data curation, and it entails the following activities:^56^

- *Data integration* The raw data entries in the dataset were derived from various research articles that use a variety of physical units to represent parameters (for example, SSA can be expressed in m^2^/g or in cm^2^/g). We maintained consistent physical units across the dataset and converted them whenever needed.
- *Outlier detection* The dataset was analysed to identify missing values, erroneous values extracted from research articles, or values that are incorrectly formatted, which could skew the results.

Once data curation is completed, the clean dataset (4538 data entries) can be used to train and test the regression algorithms.

### Regression model and metrics

Five approaches were adopted to carry out the regression of the target specific capacitance from the physicochemical features of the supercapacitors (see Supplementary Note 3 for details), namely the Ordinary Least Square Regression (OLS) method and four ML approaches: Support Vector Regression (SVR); Regression Decision Tree (DT); Random Forest Regression (RF); Extreme Gradient Boosting Regression (XGBoost).

OLS is one of the most common regression models, where the unknown parameters of linear regression are estimated by lessening the sum of the squares of the differences between the target responses of the sample data and the value foreseen by a linear function of explanatory variables^57^.

SVR is a well-established supervised machine learning approach for predicting discrete values. SVR operates on the same principle as Support Vector Machine (SVM). The primary principle of SVR is to determine the best fit line. Support vectors are the results of ideal hyperplanes, which classify unseen datasets that support hyperplanes^58^. SVM defines an optimal hyperplane as a discriminative classifier, whereas—in SVR—the best fit line is the hyperplane with the most point. The hyperplane in a two-dimensional region is a line separating into two segments wherein each segment is placed on either side. For instance, multiple line data classification can be done with two distinct datasets (*i.e.,* green and red) and used to propose an affirmative interpretation. However, selecting an optimal hyperplane is not an easy job, as it should not be noise sensitive, and the generalization of datasets should be accurate^59^. Pertinently, SVM is used to determine the optimized hyperplane that provides considerable minimum distance to the trained dataset^58,59^. SVR attempts to minimize the difference between the real and predicted values by fitting the best line under a certain threshold value. The distance between the hyperplane and the boundary line is the threshold value^60^.

DT constructs the regression or classification models based on the data features in the tree’s configuration. In a tree, every node is related to the property of a data feature. Moreover, it either predict the target value (regression) or predict the target class (classification). The closer the nodes in a tree are, the greater their influence^61^. Some benefits of the DT include the capability of handling both categorical and numerical data.

RF is an ensemble learning technique that can perform both regression and classification tasks utilizing the multiple decision trees. During training, the algorithm generates a large number of decision trees using a probabilistic scheme^62^; every tree is trained on a bootstrapped sample of the original training data and finds a randomly selected subset of the input variables to determine a split (for each node). Every tree in the RF makes its own individual prediction or casts a unit vote for the most popular class at input *x*. These predictions are then averaged in case of regression or the majority vote determines the output in case of classification^62^. The core concept is to use numerous decision trees to determine the final output rather than depending on individual decision trees.

XGBoost is one of applications of gradient boosting machines mainly designed for speed and performance in supervised learning. In supervised learning, various features in the training data are utilized to predict the target values. XGBoost applies the tree algorithms to a known dataset and categorises the data accordingly^63^. In this model, decision trees are constructed sequentially. Weights are very significant in XGBoost: they are assigned to all the independent variables which are then input into the decision tree which determines the outcomes. The weight of variables predicted wrong by the tree is increased and these variables are fed to the second decision tree. These distinct classifiers are then combined to form an efficient and precise model. XGBoost can be used for both classification and regression problems^64^.

To predict the performance of the regression models, the $n$ predicted results ($y_{i}$) were compared to the original ones ($\widehat{{y_{i} }}$) using the following metrics:^65^.

- *Root mean square error (RMSE):*
  1 $$ {\text{RMSE = }}\sqrt {\frac{1}{n}\mathop \sum \limits_{i = 1}^{n} \left( {y_{i} - \widehat{{y_{i} }}} \right)^{2} } . $$

A RMSE value closer to zero denotes a better prediction.

- *Coefficient of determination (R*^*2*^*):*
  2 $$ R^{2} = 1 - \frac{{\sum \left( {y_{i} - \widehat{{y_{i} }}} \right)^{2} }}{{\sum \left( {y_{i} - \overline{y}} \right)^{2} }}, $$

where $\overline{y}$ is the mean of $\widehat{{y_{i} }}$ values. An $R^{2}$ value closer to one represents better prediction.

- *Bias factor (*$$b^{\prime }$$*):*
  3 $$ b^{\prime } = \frac{1}{n}\mathop \sum \limits_{i = 1}^{n} \frac{{y_{i} }}{{\widehat{{y_{i} }}}}. $$

The value predicted by the model is unbiased if $b^{\prime } = 1$.

- *Mean absolute percentage error (MAPE):*
  4 $$ MAPE = \frac{100\% }{n}\mathop \sum \limits_{i = 1}^{n} \left| {\frac{{y_{i} - \widehat{{y_{i} }}}}{{y_{i} }}} \right|. $$

A MAPE value closer to zero denotes a better prediction.

## Results and discussion

### Correlation analysis

**Figure 3:**
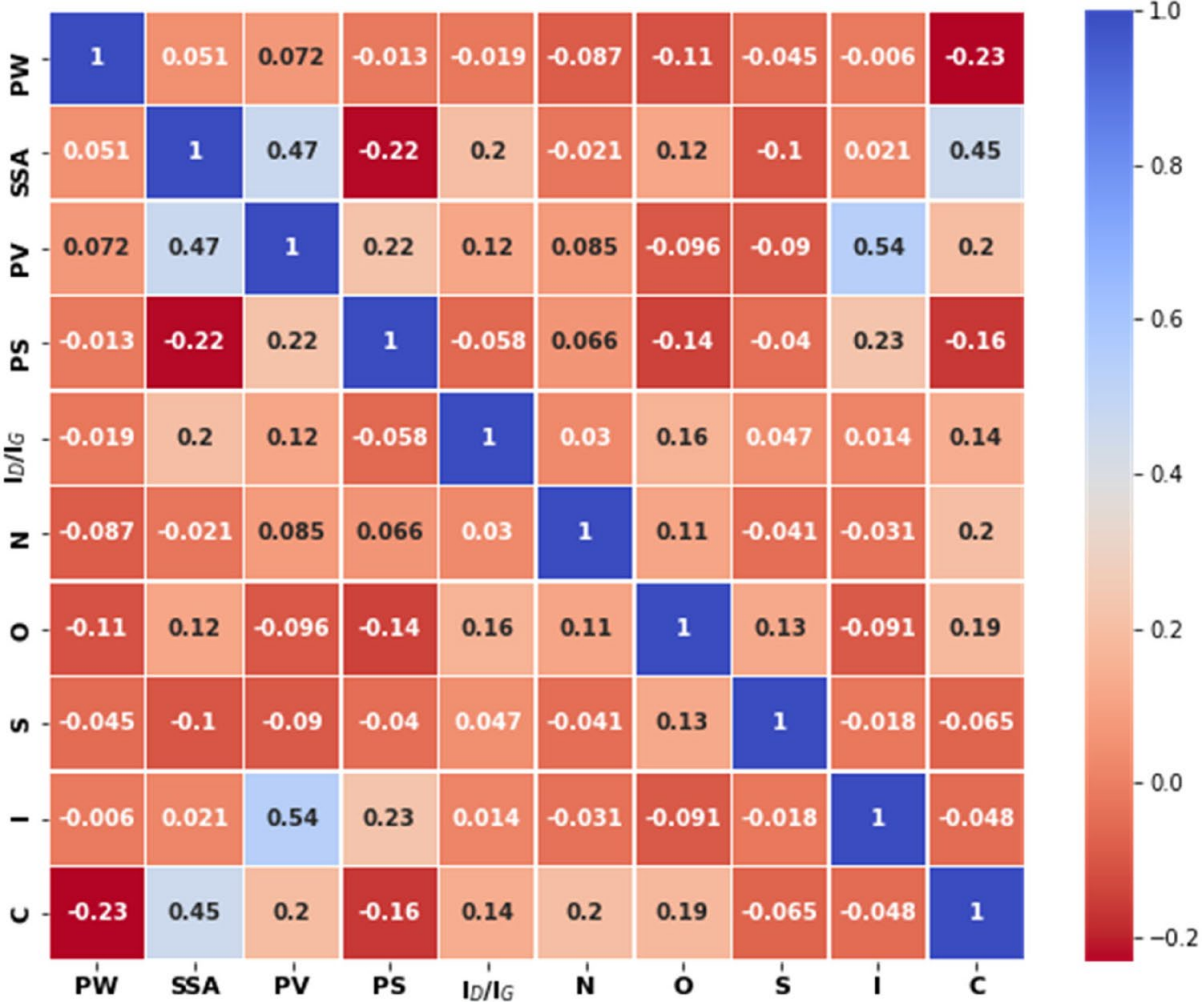
Spearman’s correlation between the physicochemical characteristics of the carbon electrodes of supercapacitors in the considered dataset.

The correlation analysis is a statistical technique used for determining the strength of a relationship between a pair of parameters (variables)^66^. To estimate the correlations between each pair of parameters, the Spearman’s rank correlation coefficient ($r_{s}$) was used, in order to encompass nonlinear relations as well^67^. The correlation (absolute values of $r_{s}$) between the various supercapacitor’s parameters (i.e., possible descriptors) and their specific capacitance (i.e*.*, figure of merit) at different intervals are presented in Table 1. This dataset analysis revealed that the specific capacitance of the supercapacitor had a moderate correlation with the SSA, a weak correlation with the nitrogen and oxygen content of the carbon electrode, and a very low or negligible correlation with the remaining parameters. These findings suggest that SSA, N%, and O% are important parameters for the enhancement of the capacitive performance of supercapacitors. In addition, the cross-correlation analysis between all considered parameters depicted in Fig. 3 shows that the physicochemical parameters of carbon electrodes are largely independent of one another, except for SSA, PV, and PS, which have a weak or a moderate geometrical intercorrelation. As a result, we can assert that the physicochemical parameters of the carbon electrode have mostly an independent effect on the supercapacitor's specific capacitance, thus they should be better considered separately from each other.

**Table 1:** Spearman's rank correlation coefficient between the specific capacitance and different features of carbon supercapacitors in the considered dataset.

$|r_{s} |$	Level	Parameters
0.0–0.19	Very weak	C versus PW, C versus PV, C versus PS, C versus I_D_/I_G_, C versus I
0.2–0.39	Weak	C versus N%; C versus O%
0.4–0.59	Moderate	C versus SSA
0.6–0.79	Strong	–
0.8–1.00	Very strong	–

### Comparison between regression methods

**Figure 4:**
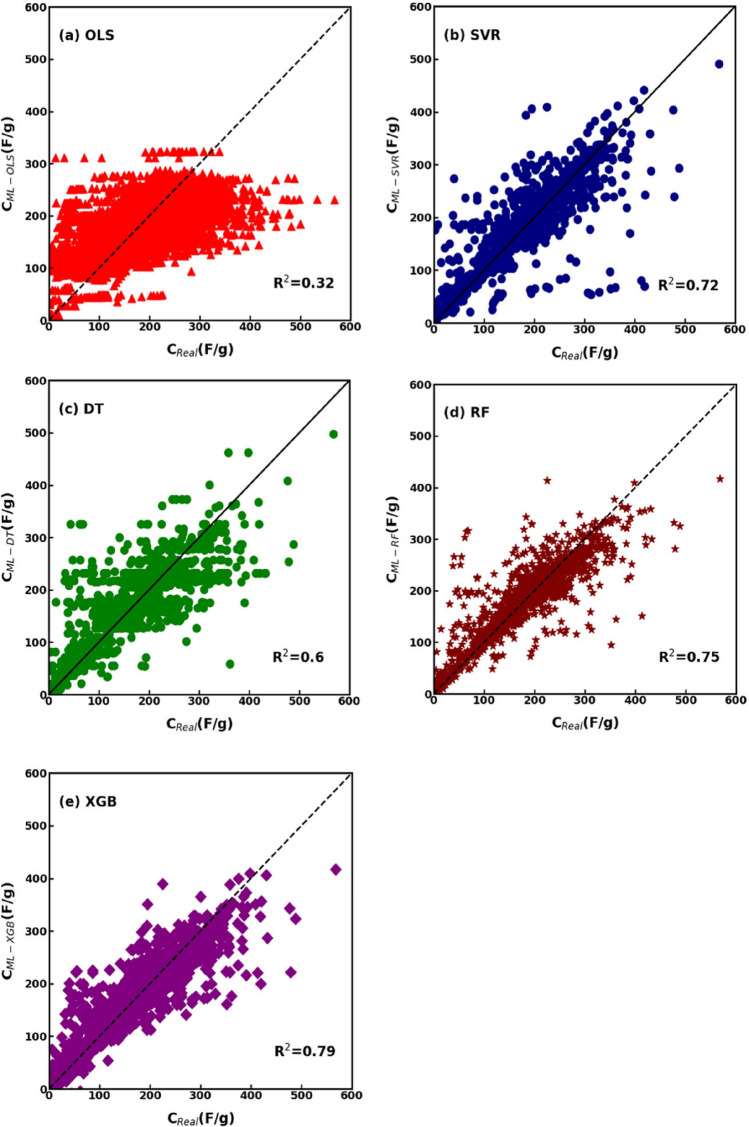
Comparison between the actual specific capacitance from literature research articles in the dataset and the predicted specific capacitance from (**a**) OLS, (**b**) SVR, (**c**) DT, (**d**) RF, and (**e**) XGBoost models.

After completing data profiling and correlation analysis, we applied five different regression models to the dataset: ordinary least square regression, support vector regression, decision tree, random forest, and extreme gradient boosting. The dataset was divided into two parts: 70% of the data were randomly selected for training the regression models and the remaining 30% for testing. The total number of data entries used for training and testing was 3176 and 1362, respectively. The nine physicochemical characteristics of the carbon electrodes of supercapacitors in the dataset were considered as independent variables, while the resulting specific capacitance as the dependent one. The results are depicted in Fig. 4 as a comparison between the real specific capacitance ($C_{Real}$) obtained from the literature articles in the dataset and the values of specific capacitance predicted by the regression models ($C_{ML}$). In each panel, the perfect match between the actual specific capacitance and the predicted one is shown via the straight diagonal line, where $C_{Real} = C_{ML}$.

As illustrated in Fig. 4a, the matching between the actual and predicted values of specific capacitance using OLS method was low, as evidenced by the significant deviation of numerous data points from the diagonal line and the *R*^*2*^ value of 0.32. Additionally, the large *RMSE* and *MAPE* values in Table 2 indicate that OLS regression achieves inferior prediction capability when compared with DT, SVR, RF, and XGBoost approaches.

**Table 2:** Performance analysis of the different regression models on the collected dataset of carbon-based supercapacitors.

Model	R^2^	RMSE	b'	MAPE
OLS	0.32	71.52	1.00	100.62
SVR	0.72	46.3	0.98	31.59
DT	0.60	55.63	1.00	36.28
RF	0.75	43.96	0.98	27.09
XGBoost	0.79	40.27	0.95	30.08

The performance analysis of the tree-based model indicates that the DT model in Fig. 4c does not accurately predict the actual specific capacitance. Instead, the SVR, RF and XGBoost models were more accurate at predicting the specific capacitance. As illustrated in Fig. 4b,d,e, most data points lie near the diagonal line, indicating prediction accuracy as supported by the *R*^*2*^ values of 0.72, 0.75 and 0.79 for the SVR, RF and XGBoost models, respectively. Moreover, other performance parameters (*RMSE*, *b′* and *MAPE*) also indicate that the SVR, RF and XGBoost models yielded superior regression capabilities when compared to the OLS and DT models. Since the performance analysis in Table 2 revealed that the XGBoost model showed the best *R*^*2*^ and *RMSE* values, only this regression was employed in the following analyses on the dataset. Notice that XGBoost showed better prediction performance than an artificial neural network as well (see Supplementary Note 4 for details).

### Influence of specific capacitance testing method

It is well established that the method of experimental testing can influence the magnitude of specific capacitance of supercapacitors. For instance, for the AC-based electrode developed by Meng et al*.*^68^ specific capacitance values of 225 F/g and 465 F/g were measured using two-electrode and three-electrode testing methods, respectively. Therefore, to investigate the effect of testing method on specific capacitance, the primary dataset generated in the current study was divided into two different subsets, wherein one contained the specific capacitance values obtained using the three-electrode method of testing (2754 data entries) and the other comprised the specific capacitance values obtained using the two-electrode method (1784 data entries). The XGBoost model was trained again on each of these two subsets of data.

**Figure 5:**
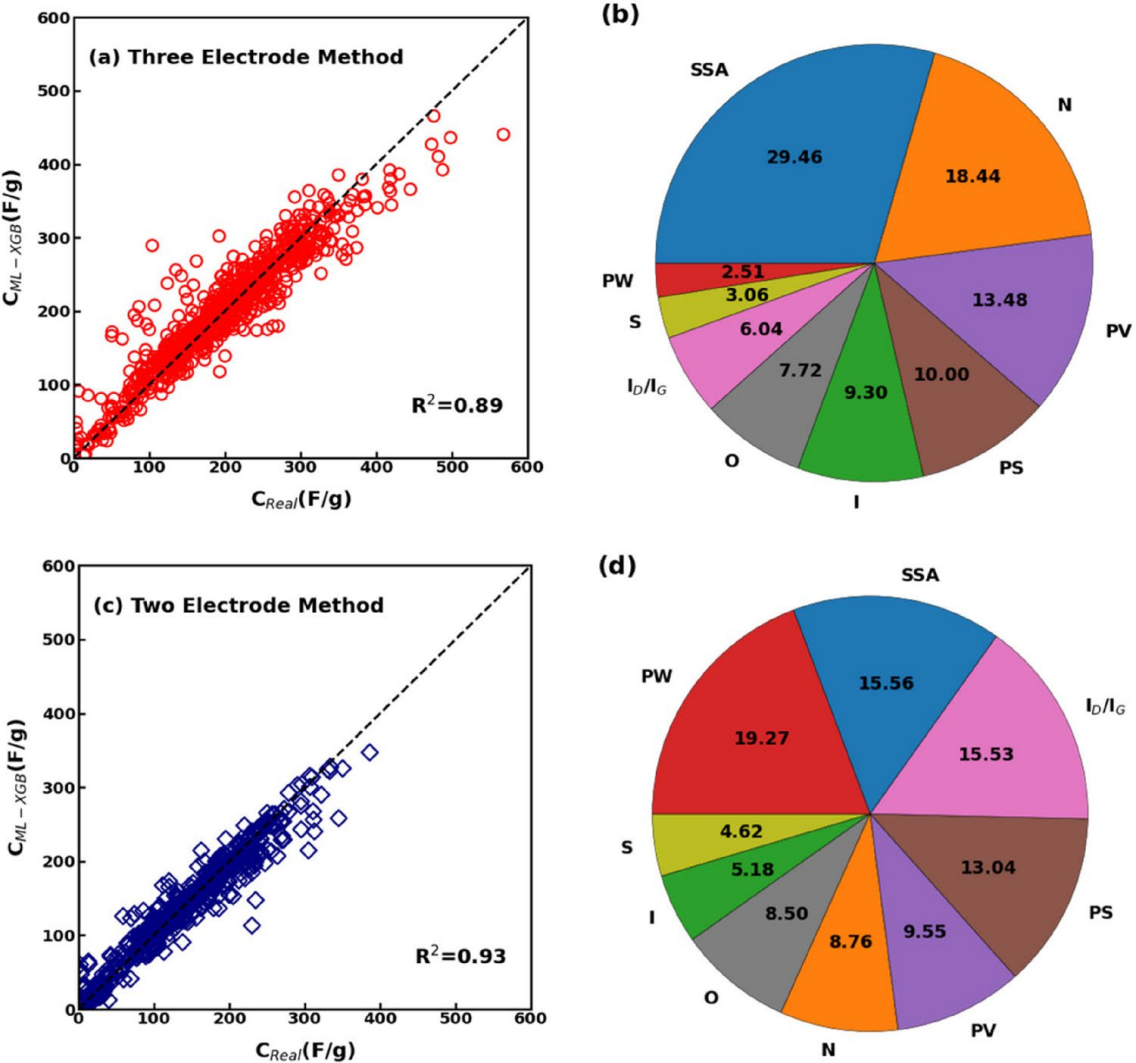
(**a**) Comparison between the predicted and actual capacitance values obtained by the three-electrode method; (**b**) feature analysis for the three-electrode method of testing. (**c**) Comparison between the predicted and actual values for the two-electrode testing method; (**d**) feature analysis for the two-electrode method of testing.

The actual specific capacitance and the predicted results by XGBoost model were found to strongly match for the three-electrode method of testing, as evident in Fig. 5a. Such good prediction capability is also highlighted by the statistical performance parameters viz*. R*^*2*^ = 0.89, *RMSE* = 28.71, *b′* = 0.98, and *MAPE* = 28.71, being improved with respect to the regression analyses on the whole dataset reported in “Comparison between regression methods” Section. Hence, the testing method has a significant effect on the specific capacitance value, being a further (categorical) variable to be considered in the prediction of specific capacitance. Thus, we investigated the significance of the different independent variable on the trained XGBoost model, which indicates how the physicochemical parameters of carbon electrodes influence the specific capacitance. Figure 5b depicts the feature importance analysis for the three-electrode testing method, where higher shares are associated to more influence of variables on specific capacitance: the SSA, heteroatom doping (N%), and PV were found to be the major factors influencing the specific capacitance.

The correlation between the actual and predicted specific capacitance for the datasets obtained using the two-electrode method of testing is shown in Fig. 5c, instead. Again, the regression accuracy is improved by considering the subset of data measured with two-electrode method rather than the whole dataset. In fact, the performance parameters for the XGBoost regression of the two-electrode dataset are *R*^*2*^ = 0.93, *RMSE* = 19.45, *b′* = 0.989, and *MAPE* = 31.07, thus better with respect to the analyses carried out on the whole dataset. In this case, the PW, SSA, and the I_D_/I_G_ ratio of the carbon electrode were found to contribute most towards enhancing the supercapacitors' specific capacitance, as observed from the feature analysis in Fig. 5d. Interestingly, the SSA is found as an influential physicochemical characteristic of supercapacitors in both testing methods, while small discrepancies emerge for the other variables. PW is found as a relevant parameter of specific capacitance only in case of two-electrode measures, thus appearing as a possible descriptor able to discriminate between the adopted method of testing.

### Influence of electrolyte

**Figure 6:**
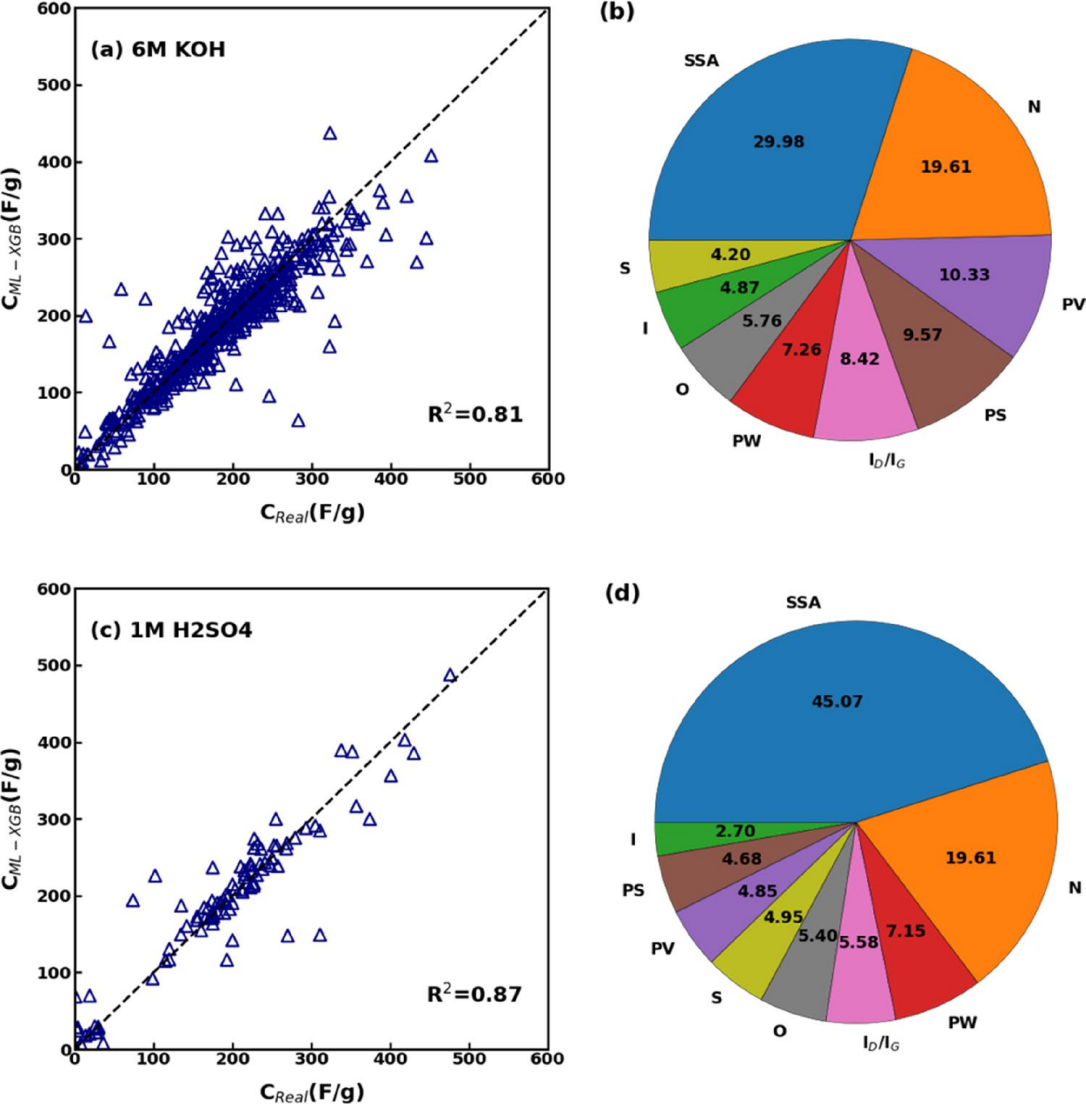
(**a**) Comparison between predicted and actual specific capacitance and (**b**) feature analysis for the subset of data having 6 M KOH electrolyte. (**c**) Comparison between predicted and actual specific capacitance and (**d**) feature analysis for the subset of data having 1 M H_2_SO_4_ electrolyte.

The specific capacitance of a supercapacitor is determined not only by the physicochemical behaviour of the electrode material and the testing method, but also by the type of electrolyte used. For instance, Zhou et al*.*^52^ synthesised hierarchical nitrogen-doped porous carbon and demonstrated a specific capacitance of 339 F/g in 6 M KOH and 282 F/g in 1 M H_2_SO_4_, respectively, at a current density of 0.5 A/g. Thus, to decouple the effect of different electrolytes from our analyses, we considered configurations with either 6 M KOH or 1 M H_2_SO_4_. Consequently, we extracted only data entries characterized by 6 M KOH (2819 entries) and 1 M H_2_SO_4_ (471 entries) from the overall dataset. In these cases, 80% of dataset was considered for training and 20% for testing the XGBoost model. The accuracy of the obtained regression is corroborated by the improved statistics of XGBoost model fitting for the 6 M KOH electrolyte (R^2^ = 0.81, RMSE = 33.86, b′ = 1.01, and MAPE = 16.75) and the 1 M H_2_SO_4_ electrolyte (R^2^ = 0.87, RMSE = 36.48.44, b′ = 0.98, and MAPE = 116.24), as depicted in Fig. 6a,c. The feature analyses carried out on supercapacitors with 6 M KOH and 1 M H_2_SO_4_ electrolytes demonstrate that the SSA, nitrogen doping, and PV were the major contributors to the capacitive performance in the 6 M KOH electrolyte, whereas the SSA, nitrogen doping, and PW in the 1 M H_2_SO_4_ electrolyte, as evident from Fig. 6b,d.

**Figure 7:**
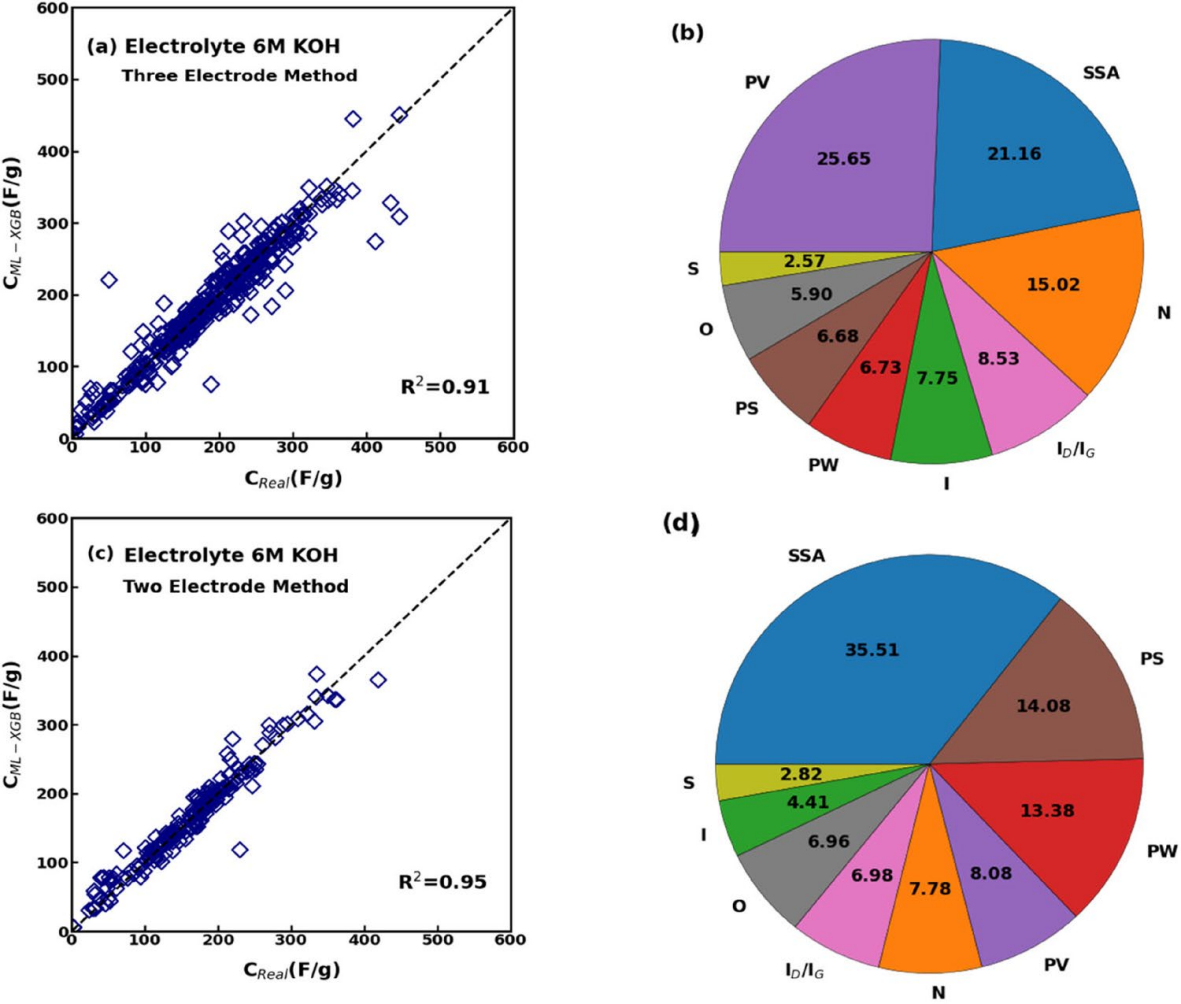
(**a**) Comparison between predicted and actual specific capacitance and (**b**) feature analysis for the subset of data having 6 M KOH electrolyte and measured by three-electrode method. (**c**) Comparison between predicted and actual specific capacitance and (**d**) feature analysis for the subset of data having 6 M KOH electrolyte and measured by two-electrode method.

Due to the limited data entries for the 1 M H_2_SO_4_ electrolyte, then we considered only the aqueous electrolyte 6 M KOH—which has also the additional benefits of being inexpensive, safe, and with a high dielectric constant and specific capacitance—to discriminating again between two-electrode and three-electrode testing methods. Refining the datasets considering a specific electrolyte (6 M KOH) and method of testing further improved the regression performance with respect to results in “Comparison between regression methods” Section (overall dataset) and Influence of specific capacitance testing method (datasets separated for three- and two-electrode testing methods). This is evident from Fig. 7a,b, where most data points are located near the diagonal line, indicating a strong correlation between the actual and predicted specific capacitance values. Furthermore, the accuracy of regression is corroborated by the improved statistics of XGBoost model fitting for both the two- (*R*^*2*^ = 0.95, *RMSE* = 16.56, *b′* = 0.97, and *MAPE* = 10.15) and the three-electrode method (*R*^*2*^ = 0.91, *RMSE* = 24.08, *b′* = 0.985, and *MAPE* = 12.88). Similarly to Fig. 5, the feature analysis carried out on supercapacitors with 6 M KOH electrolyte demonstrate that the SSA, PV, and nitrogen doping were the major contributors to the capacitive performance in the three-electrode testing method (Fig. 7b), whereas the PW, PS, and SSA in the two-electrode one (Fig. 7d). Hence, the regression performed on the limited dataset of supercapacitors with 6 M KOH electrolyte does not change the relative influence of physicochemical characteristics of supercapacitors on their performance discussed in “Influence of specific capacitance testing method”, therefore showing the robustness of the feature analysis.

Notice that, due to limited data entries for different concentrations of electrolyte in the current database (1 M KOH has 12 entries, 2 M KOH has 169 entries and 3 M KOH has 132 entries), we could not train a robust XGBoost model specifically dedicated to exploring also this effect on the specific capacitance of supercapacitors.

### Influence of carbon electrode structure

Carbon exists in various allotropic forms with distinct morphologies and physicochemical properties. Activated carbon, hierarchical porous carbon, heteroatom doped porous carbon and graphene derived carbon are mainly employed for carbon electrodes of supercapacitors. The different morphological forms of these carbon allotropes may affect the specific capacitance of supercapacitors^28^. ACs possess a large SSA and pore volume, thus easing the accumulation of static charges at the electrode surface and the resulting specific capacitance. HPC electrodes, instead, contain pores in a wide range of length scales (from micro to macro). The presence of macropores in HPC allows high-rate ion transport and acts as an ion reservoir. HA carbon electrodes are generally obtained from AC incorporated with heteroatoms (N, O, S, P), which enhance the wettability and electronic conductivity of the base material. Graphene shows also high SSA and electrical conductivity.

**Figure 8:**
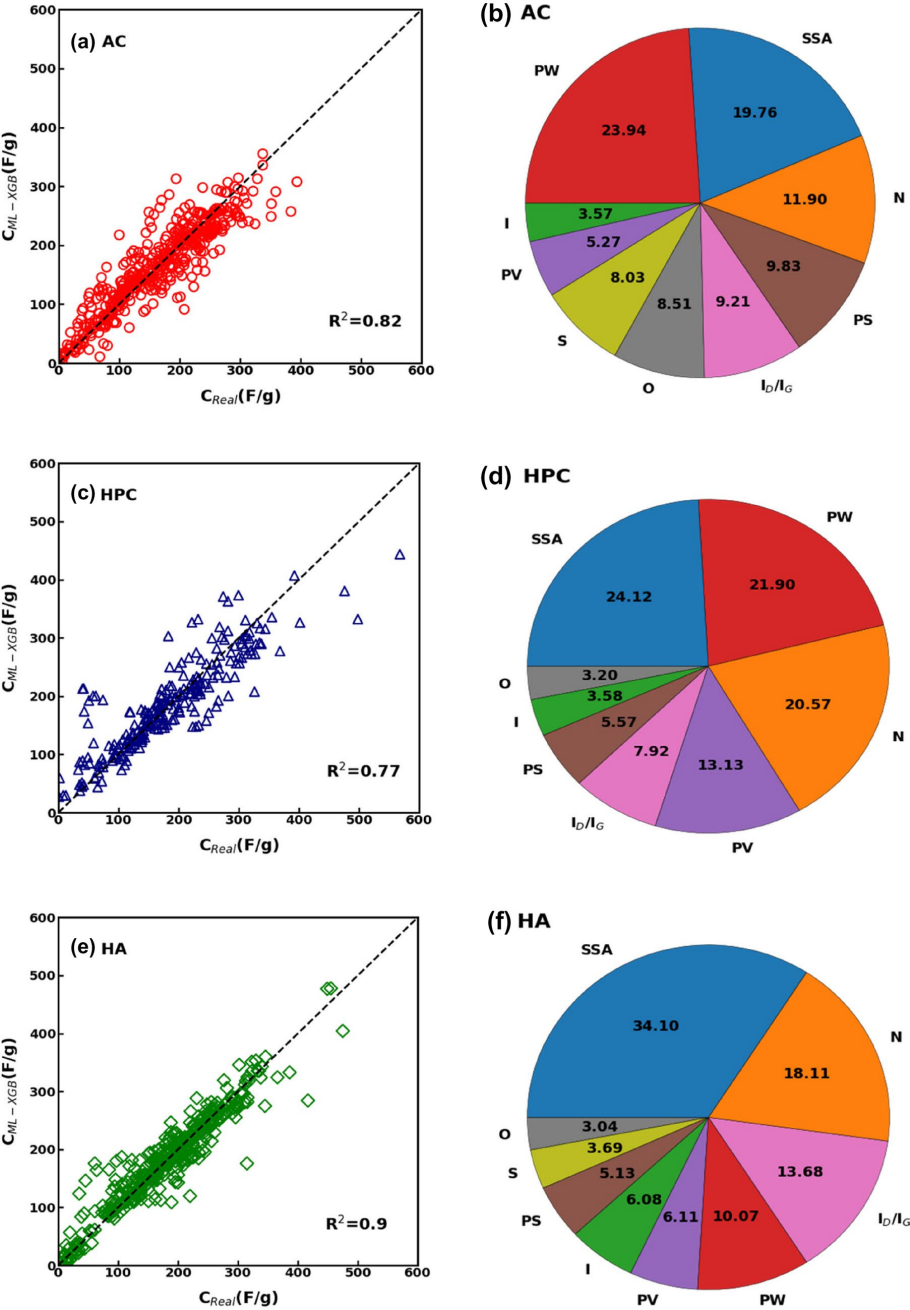
(**a**) Comparison between the predicted and actual specific capacitance and (**b**) feature analysis for the activated carbon electrodes. (**c**) Comparison between the predicted and actual specific capacitance and (**d**) feature analysis for the hierarchal porous carbon electrodes. (**e**) Comparison between the predicted and actual specific capacitance and (**f**) feature analysis for the heteroatom-doped carbon electrodes.

Therefore, we further split our dataset according to the type of carbon materials used in the construction of the electrodes. AC, HPC, and heteroatom (HA)-doped electrodes were differentiated to generate separate datasets, and XGBoost trained to best match the capacitive behaviour of supercapacitors made of specific carbon structures. Notice that, in this case, datasets have not been subdivided according to different testing methods or electrolytes, since the limited number of entries available for some classes of carbon structures did not allow a robust training of the regressor. Figures 8a,c,e compare the predicted and actual values of specific capacitance for the three types of considered carbon structures, highlighting a good match especially for HA ones (correlation statistics are detailed in Table 3). The feature analysis is done also in this case: Fig. 8b,d,f identify as most influential physicochemical features a combination of parameters previously found for two- and three-electrode testing methods, such as PW, SSA, and N%.

**Table 3:** Performance analysis of the trained XGBoost models to relate the physicochemical features of carbon electrodes with different structures and the measured specific capacitance.

Electrode type	R^2^	RMSE	*b′*	MAPE
AC	0.82	33.85	1.003	23.104
HPC	0.77	44.13	0.97	49.543
HA	0.9	26.78	0.98	16.276

Overall, the current study focused mainly on the results obtained by applying XGBoost regressors, as their accuracy was shown to be superior to that of other ML models. As a result of differentiating datasets according to the testing method, electrolyte type or morphology of the carbon electrode material, the accuracy of trained XGBoost models was further improved (see *R*^*2*^ values), while the most relevant physicochemical features identified for these different categorical variables. Considering all the feature analyses shown in Figs. 4, 5, 6, 7, and 8, SSA is by far the dominant physicochemical characteristic of electrodes in determining the specific capacitance of the supercapacitors in the dataset, followed by N% and PW (which appears to be particularly influent when two-electrode methods are employed for the measure). PV, I_D_/I_G_ and PS follow with decreasing importance, while I, O% and S% result to be the least influential features.

## Conclusions

ML models such as OLS regression, SVR, DT, RF, and XGBoost were used to predict the influence of various physicochemical parameters and categorical variables of carbon-based electrode materials on the capacitive performance of an ELDC supercapacitor. First, a dataset was developed by extracting information from 147 experimental research articles on carbon-based electrode supercapacitors. This included the presence of defects, the pore volume, pore surface, current density, surface specific area, potential window, nitrogen, oxygen, and sulphur content in carbon-based electrode materials. Categorical variables such as the testing method, electrolyte type or morphology of the carbon electrode material were also considered. These data entries (4538) were fed into five regression models, prior to which the dataset was curated to achieve consistent physical units and outlier detection. Subsequently, the Spearman’s rank correlation coefficient was used to determine the correlation between each pair of parameters, which suggested that all the available physicochemical parameters were not dependent from each other. For training the regression models, the datasets were divided into a 70:30 ratio for training and testing, respectively. Correlations between the actual specific capacitance and the predicted specific capacitance of the five models are ranked as follows: XGBoost > RF > SVR > DT > OLS, thus showing a superior regression performance by ML algorithms.

Additionally, we used the XGBoost model to predict the effect of the testing method (two- and three-electrode method) on the specific capacitance of supercapacitors. This resulted in acceptable performance parameters for both the testing methods. Furthermore, in the three-electrode method, SSA, N%, and PV were identified as the major contributors, whereas in the two-electrode method SSA, PW, and I_D_/I_G_ were observed to significantly influence the capacitive performance. To comprehend the impact of the electrolyte on the specific capacitance, we further extracted datasets having 6 M KOH in the two-electrode and three-electrode testing methods. The performance parameters obtained using the XGBoost method suggested improved statistics for both the testing methods. As a result, the PV, SSA, and N% were identified as the significant contributors in the three-electrode method, whereas SSA, PS, and PW were confirmed to be the significant contributors in the two-electrode method. Finally, using the XGBoost model, we determined the various physicochemical characteristics according to the type of electrode materials used for the construction of the electrode that affect the specific capacitance of supercapacitor. The heteroatom (HA)-doped carbon exhibited a better regression in comparison to the AC and HPC. Overall, SSA appears as the most influential physicochemical characteristic of electrodes in determining the specific capacitance, followed by N% and PW. PV, I_D_/I_G_ and PS have a decreasing importance, while I, O% and S% the least.

We highlight that the imperfect matching between the currently trained ML models and the considered experiments may be also due to different experimental conditions during the supercapacitor testing. For instance, electrode conditioning before property measurements usually involves extensive charge–discharge cycling or holding the electrode for some time at an elevated temperature and potential, and it typically decreases or even removes certain surface functional groups, which in turn improves storage stability. Unfortunately, not all researchers perform electrode conditioning before measuring properties or even identify possible current leakages when reporting measurements. This study, however, is not intended to replace modelling or experimental analyses, but rather to provide a preliminary support in the design and development of new supercapacitors, with the possibility to re-train and thus refine the presented ML models as soon as further data and descriptors will become available in the literature (e.g., extensive data on carbon material percentage in the electrode, cf. Supplementary Note 5).

In conclusion, the current study demonstrated the successful utilization of a data-driven method to predict the material performance for supercapacitor applications and revealed the most significant parameters that affect the specific capacitance of EDLCs. In perspective, the curated dataset developed and shared in this work may facilitate further analyses and potential optimization of carbon-based electrodes in different electrochemical applications. To ease the exploitation of the trained models (see Supplementary Note 6) by experimentalists, we developed a software with a graphical user interface (SUPERCAPs^69^) that allows to easily provide with an estimate of the specific capacitance of carbon-based supercapacitors knowing the physicochemical characteristics and structure of carbon electrodes, the testing method, and electrolyte.

## Supplementary Information

Supplementary Information.

## Data Availability

All data collected and analyzed during this study are included in this published article, the related supplementary information files and Zenodo archive (10.5281/zenodo.7346943).
