# Supplementary material for: The impact of physicochemical features of carbon electrodes on the capacitive performance of supercapacitors: a machine learning approach
Source: Sci Rep. 2023 Apr 20;13:6494. doi: 10.1038/s41598-023-33524-1 (PMC10119157; doi:10.1038/s41598-023-33524-1)
Supplement: Supplementary file 1 — Supplementary Information. [file 41598_2023_33524_MOESM1_ESM.pdf]

The impact of physicochemical features of carbon electrodes on the capacitive performance of supercapacitors: A machine learning approach

# - Supplementary Information -

Sachit Mishra[a,b,1], Rajat Srivastava[a,c,1], Atta Muhammad[a,d], Amit Amit[a], Eliodoro Chiavazzo[a], Matteo Fasano[a,*], and Pietro Asinari[a,e]

[a] Department of Energy “Galileo Ferraris”, Politecnico di Torino, Corso Duca degli Abruzzi 24, 10129 Torino, Italy.

[b] IMDEA Network Institute, Universidad Carlos III de Madrid, Avda del Mar Mediterraneo 22, 28918 Madrid, Spain.

[c] Department of Engineering for Innovation, University of Salento, Piazza Tancredi 7, 73100 Lecce, Italy.

[d] Department of Mechanical Engineering, Mehran University of Engineering and Technology, SZAB Campus, Khairpur Mir’s 66020, Sindh, Pakistan.

[e] Istituto Nazionale di Ricerca Metrologica, Strada delle Cacce 91, 10135 Torino, Italy.

* Corresponding author.

Email address: matteo.fasano@polito.it (Matteo Fasano).

[1] *Equal contributors.*

Supplementary Note 1: Review on carbon electrodes

Porous and activated porous carbon

Because of their high specific surface area (SSA), improved electrical conductivity, adjustable pore-sizes, electrochemical stability, and low cost, activated porous carbons (AC) offer significant promise for use as the electrode [1-4]. These properties make AC an excellent material for a variety of applications, including water purification, gas separation and storage, and electrode materials for capacitors, fuel cells and batteries. Porous/activated carbons are prepared by the pyrolysis of petroleum coke and coal followed by physical/chemical activation [5]. In recent years, synthesising AC from fossil raw materials has been highly discouraged and several efforts were made to find sustainable and green sources of raw materials for the AC preparation. In this direction, biological matters such as roots, flowers, stems, leaves, fungi fruits, and animal body parts etc., have been adopted as resources for the synthesis of AC [6]. Additionally, several methodologies have been used to synthesise the AC from the biomass precursor including pyrolysis, hydrothermal carbonization, mechano-chemical, hard- and soft-templating [7].

In pyrolysis, the biomass is heated at an elevated temperature (T = 300–1000 °C) under the presence of inert gas (e.g., nitrogen, argon). The conversion of biomass into AC involves several steps including removal of moisture (T<100 °C), degradation of cellulose and hemicellulose (in temperature range 200 °C<T<500 °C), and the decomposition of lignin (T>500 °C). The carbonization of biomass removes all the volatile materials, and a major part of the residual solid is carbon [8]. Additionally, the application of pyrolysis-derived carbons (DPCs) in energy storage devices depends on their pore morphology and physicochemical characteristics.

Usually, DPCs have a low SSA and unsuitable pore morphology for their application in supercapacitors. Thus, an activation process (physical or chemical activation) is needed to enhance the physicochemical characteristics of DPC. In the case of physical activation, the carbon precursor is carbonized at a temperature (T<800 °C) followed by an activation process in the existence of activating agents such as air, CO_2, and steam at an elevated temperature. The chemical activation process requires the mixing of carbon precursor with an activating agent at a suitable temperature [9]. Because of its lower activation temperature, high yield, and generation of microporous carbon with a large SSA, potassium hydroxide (KOH) is used as one of the most common chemical activating agents.

In hydrothermal carbonization (HTC), the carbonization process of biomass occurs at natural conditions, i.e., in presence of an aqueous medium at mild temperature (130–250 °C) and pressure (0.1 MPa). This process is very complex and usually contains five steps: hydrolysis, dehydration, decarboxylation, polymerization, and aromatization [10]. Additionally, in HTC processes, the nature and morphology of the AC can be tuned by varying the temperature under mild processing conditions [10].

In general, ACs possess a large SSA (> 1000 m^2/g) and pore-volume (> 0.5 cm^3/g), which are critical characteristics because the accumulation of static charges at the electrode surface determines the charge storage capacity of the supercapacitor. However, in practical observation, the capacitance of the ACs electrode supercapacitor is only about 10–20% of its

theoretical capacitance [11]. This is either due to the presence of inaccessible micropores or very large pores generated during carbon activation. Therefore, the high capacitive performance of the supercapacitor depends on the characteristics of electrode materials such as large SSA and optimal pore-size distributions to ease the transport of electrolyte ions within the pores [11].

The cost of AC hinders its application in a supercapacitor as an electrode material. Recent studies reported that the AC can be successfully synthesised using various biological sources and wastes such as pitaya peel [12], corncob residue [13], water bamboo [14], a harmful aquatic plant (*Altemanthera philoxeroides*) [15], pumpkin [16], pomelo peel [17]. They are cost-effective, environmentally friendly, in abundance, and renewable [18]. One such low-cost bio-waste is rice husk produced during the processing of rice. The annual production of rice husk is about 120 million tonnes all over the world [19] and its disposal is a serious environmental concern as the most common disposal method is the open-air burning of rice husk in an uncontrolled environment, which releases a large amount of carbon monoxide (CO) and carbon dioxide (CO_2) [20]. Cellulose, hemicellulose, silica, lignin, and moisture are the main constituent of the rice husk [21].

Guo *et al.* [22] used rice husk to synthesise AC with high SSA (ranging from 1392–2721 m^2/g) by using alkaline hydroxide (KOH and NaOH) activation. They reported that the EDLC with rice husk AC electrode could achieve the specific capacitance of 210 F/g in the KCL solution. Furthermore, a high-performance supercapacitor using rice husk derived AC electrode developed by He *et al.* [23] obtained a capacitance of 245 F/g in a current density of 0.05 A/g, along with a slight decrement in the charge storage capacity (233 F/g) under increasing current densities (2 A/g). Wang *et al.* [24] also synthesised a AC electrode from hydrochar derived rice husk by KOH activation, which yielded a high specific capacitance of 312 F/g due to the high SSA (3362 m^2/g) of the activated rice husk hydrochar electrode. Additionally, Chen *et al.* [25] synthesised a AC electrode for EDLC supercapacitor with a SSA of approximately 4000 m^2/g, which obtained a specific capacitance of 368 F/g in a 6 M KOH electrolyte.

Similar to rice husk, corn stalk core is also a bio-waste that shows the possibility for the generation of AC electrodes for supercapacitors. The corn stalk core contains natural pores distributed in a sponge-like structure that are suitable for preparing the high surface area and AC electrode raw material. Yu *et al.* [26] prepared ACs electrode using corn stalk core with a high surface area (2349.89 m^2/g) and determined the specific capacitance of 140 F/g (at 1 A/g current density). Additionally, Wang *et al.* [27] reported the conversion of corncobs to activated carbon using chemical activation for application in a supercapacitor. The resultant ACs exhibited a high SSA of 3054 m^2/g, and specific capacitances of 401.6 F/g and 328.4 F/g in 0.5 M H_2SO_4 and 6 M KOH electrolyte, respectively, at a current density 0.5 A/g. Karnan *et al.* [28] fabricated a supercapacitor device using an activated carbon electrode derived from corncobs and an ionic liquid electrolyte. With just a ten-second charge, the device could power a LED light for more than 4 minutes. The electrochemical performance of activated carbon (AC) electrodes in 0.5 M H_2SO_4 electrolyte also revealed the high capacity of the corncob electrode with a specific capacitance of 390 F/g at 0.5 A/g [28]. Several authors also developed activated carbon (AC) electrodes from corn-based biomass such as corn straw, corn gluten, popcorn, etc. with improved SSA as well as reasonable electrochemical performance [29-31].

In a recent study, Rajabathar *et al.* [32] prepared a porous AC nanostructured electrode using jackfruit peel waste (JFPW), which showed outstanding electrochemical performance with a specific capacitance of 320 F/g at low current density (1 A/g) in 1 M Na_2SO_4 electrolyte. They also reported that AC electrodes derived from jackfruit retained a high specific capacitance 274 F/g even at a high current density (5 A/g). Similar evidence of a high-performance supercapacitor was also reported using AC electrode prepared from pitaya peel having a specific capacitance of 255 F/g at a current density 1 A/g and 96.4% retention capacity at 5 A/g with excellent stability in 6 M KOH electrolyte [12]. Additionally, Lin *et al.* [17] synthesised hemicellulose AC raw material from pomelo peel for an electrode with a high SSA of 1361 m^2/g and excellent charge storage capacity of 302.4 F/g at a current density of 0.5 A/g.

Three-dimensional sakura-based activated carbons have also been utilized as the raw material for the electrode of supercapacitor. The electrochemical performance was analysed in a three-electrode method of testing with 6 M KOH electrolyte, reporting a specific capacitance of 265.8 F/g for sakura-based active carbon at a current density 0.2 A/g [33]. Chang *et al*. also synthesised activated ACs using paulownia flower as the precursor, which offered 297.1 F/g specific capacitance at 1 A/g in 1 M H_2SO_4 electrolyte [34]. Zhang *et al.* [35] reported the conversion of bamboo through carbonization and chemical activation into activated porous carbon for supercapacitor electrodes with a high specific capacitance of 293 F/g at 0.5 A/g current density. Wang *et al.* [36] modified commercially available coconut shell-based AC (CSAC) through H_2O plasma resulting in an environmentally friendly method to generate electrode material (HCSAC) with enhanced specific capacitance and retention capability compared to its precursor CSAC.

Activated carbon-based supercapacitor electrodes have also been synthesised using a low cost, highly porous willow-wood. The resultant ACs exhibit a high surface area (2793 m^2/g), pore-volume (1.45 cm^3/g) and the presence of both micro- and meso- pores that are favourable for the energy storage [37]. The obtained AC electrode supercapacitor also showed a magnificent electrochemical performance having a specific capacitance of 394 F/g at a current density of 1.0 A/g in an aqueous electrolyte (6 M KOH) [37].

One socio-economic and environmental concern worldwide is to get rid of seaweed (*Ascophyllum nodosum*) blooms, which is in abundance in northern oceans. Chemically activated biocarbon electrode derived from *Ascophyllum nodosum* can also be used in supercapacitors. Perez-Salcedo *et al.* [38] synthesised an activated biocarbon electrode derived from *Ascophyllum nodosum* for supercapacitors with a capacitance of 207.3 F/g at current density (0.5 A/g), excellent stability and retention capability.

Hierarchical porous carbon

The hierarchical porous carbon (HPC) material contains pores in a wide range of length scales that are missing in the conventional porous material. The HPC contains pores in the ranges of macro- (>50 nm), meso- (2–50 nm), and micro- (<2 nm) scales. The presence of macropores in HPC allows high-rate ion transport and acts as an ion reservoir. Furthermore, the interconnected mesopores provide low resistance pathways for the diffusion of ions, and the high SSA of micropores, which enhances the adsorption of ions at the pore surface [39]. These unique properties of HPC material gained recent interest in the selection of electrode material for supercapacitors. The development of hierarchical porous structure from carbon

material requires templating techniques. There are two types of templating: soft template; hard template. The soft templates were used as a substance for self-assembly; whereas the hard template method consists of the three steps: 1) impregnating the pre-synthesised template; 2) carbonization; 3) peeling off the hard template. It is followed by the carbon conversion process (carbonization) and etching [39].

However, these methods are complex, time demanding, and expensive. In addition, it is hard to regulate porosity that creates a serious obstruction in the usage and large-scale production of HPC. Thus, there is a special need for the development of an easy, inexpensive, and eco-friendly procedure for the synthesis of HPC materials.

The synthesis of AC material from natural biomass such as cotton is an eco-friendly and economical approach for the development of electrodes for supercapacitors. The bio swelling of cotton fibres under the influence of NaOH/urea enables the formation of HPC fibres with improved surface characteristics [40]. The cotton fibre derived HPC fibres electrode material possess a high SSA (584.49 m^2/g), along with favourable pore morphologies that enhance the specific capacitance (221.7 F/g at 0.3 A/g) [40].

Bagasse (a biomass-waste from sugar industries) were used to synthesise carbonaceous material for the absorption of heavy metal ions, organic pollutants, and it finds its applicability in energy storage application. However, the carbonaceous material derived from bagasse contains narrow pore-size distribution and insufficient SSA that restricts its applicability in supercapacitors. Feng *et al.* [41] developed a simple method for the synthesis of HPC from bagasse using sewage sludge assisted hydrothermal carbonization with KOH activation. This process is a cheap and efficient way to regulate the porosity and structure of HPC and results in an excellent supercapacitive performance. The bagasse-derived hierarchical structured carbon (BDHSC) electrode supercapacitor possesses 320 F/g capacity at 0.5 A/g current density with good cyclic stability. Zhou *et al.* [42] synthesised the nitrogen-doped porous carbons (HNPCs) from biomass precursor cellulose carbamate with tuneable pore structures and ultrahigh SSAs via simultaneous carbonization and activation using a facile one-pot approach. They exhibited ultrahigh specific surface area (3700 m^2/g), high pore-volume (3.60 cm^3/g) and high-level nitrogen doping (7.7%). In three-electrode system, HNPCs showed specific capacitance of 339 F/g with 6 M KOH electrolyte and 282 F/g with 1 M H_2SO_4 electrolyte at a current density of 0.5 A/g, whereas in two-electrode system it exhibited a high specific capacitance of 289 F/g at 0.5 A/g.

Gou *et al.* [43] prepared a HPC material for the electrode of the supercapacitor from wheat straw cellulosic foam with high SSA of 772 m^2/g after KOH activation and micropores ranging from 1.05-1.74 nm with 6 M KOH electrolyte. The high porosity provides the better migration of the ions in an electrolyte, thus enhancing the electrochemical characteristics of the capacitors. In three-electrode system, they obtained a capacitance of 226.2 F/g at a current density of 0.5 A/g. This provides a method for obtaining electrode materials from the cheap and easily available material wheat straws.

In a recent study, Zhao *et al.* [44] prepared N-O co-doped AC from low-cost, sodium alginate particles for the development of supercapacitors. They obtain the N-O co-doped AC from the crosslinking of SA beads with diammonium chains with the help of electrostatic interaction between ammonium cations and carboxylate groups of SA chains. Both the species (N-O) and concentration of diammonium chains strongly affected the electrochemical

characteristics of NO co-doped AC. They obtained capacitance performance of 269.0 F/g at current density of 1 A/g.

Heteroatom doped porous carbon

Besides the SSA and pore-volume, there are also several other factors that influence performance such as surface functional groups and conductivity (pseudocapacitance and overall electric capacity). These can be achieved by the appropriating carbon with heteroatoms (nitrogen, oxygen, sulphur, etc.). By doing so, it not only enhances the wettability but also improves electronic conductivity of AC [45]. Among these heteroatoms (N, O, S, P), sulphur is the highest reactive element. Sulphur doping of carbonaceous material increases its band-gap that enhances the electron donor properties and change in the electronic density of state. Sulphur doping also increases wettability, which in turn decreases the diffusion resistance that occurs between the electrode and electrolyte ions [46].

Li *et al.* [47] used willow catkin to develop porous carbon nanosheets (PCNs) from pyrolytic and activation approaches, followed by the co-doping of nitrogen and sulphur. The N-S co-doped carbon nanosheets electrode supercapacitor possesses 298 F/g capacity at 0.5 A/g current density and 298 F/g capacity at 0.5 A/g with green and low-cost materials for electrode of supercapacitor. Wang *et al.* [45] reported a porous nitrogen self-doped carbon material with layer structure for high-performance supercapacitors. It was derived from the by-product of the pig-farming industry porcine bladders. It possesses C, N and O elements in abundance. Combining carbonization and KOH activation processes yielded the nitrogen self-doped layered AC. KOH dosage can be changed to adjust the amount of N and pore structure. It has outstanding electrochemical characteristics including high specific capacitance of 322.5 F/g and good cycle stability during 5000 cycles.

Kim *et al.* [43] reported a straightforward method for biomass-derived AC with a high surface area and heteroatom doping. It involves exothermic pyrolysis of Mg/K/MgK-nitrate-urea-cellulose mixture followed by a high temperature carbonization and washing treatment. The produced N-doped AC material shows specific capacitance of 279 F/g at 1 A/g in 6 M KOH electrolyte in the two-electrode method of testing. Wan *et al.* [48] prepared three AC from lotus pollen for supercapacitor activated with ZnCl2, $FeCl_3$, and $CuCl_2$. AC obtained by $CuCl_2$ activation exhibits higher surface area, more porous and higher heteroatom doped than traditional activated $ZnCl_2$ or $FeCl_3$ AC.

Demir *et al.* [49] reported a method for the sustainable and economic transformation of waste product lignin (by-product of paper and pulp industry) into heteroatom doped AC used for electrodes of supercapacitor and CO_2 capture applications. The synthesis process involves carbonization and chemical activation. The synthesised AC contains 2.5 to 5.6 wt.% nitrogen and 54 wt.% oxygen in its final structure. It possesses a high surface area 1788 to 2957 m^2/g, capacitance of 372 F/g and excellent cyclic stability over 30,000 cycles in 1 KOH. Razmjooei *et al.* [50] developed AC from the most available human waste, urine. It started with the removal of mineral salt from urine carbon (URC), which makes it more porous followed by heteroatom doping (N, S and P). The combined effect of surface properties and porous structure makes it feasible for energy storage applications. It exhibits 1040.5 m^2/g surface area, good conductivity and heteroatom doping of N, S and P exhibiting capacitance of 166 F/g at 0.5 A/g in a three-electrode method of testing.

Graphene derived carbon

In perspective, graphene is the most promising electrode material for various energy storage applications in particular supercapacitors due to a high electrical conductivity, high SSA, and excellent mechanical strength [51]. Its porous structure also facilitates charge transport in the supercapacitor. These exceptional properties of graphene make it a suitable candidate for the supercapacitor electrode.

The SSA of graphene is highly tuneable according to the required supercapacitor electrode for energy storage applications. Also, the presence of highly movable free pi (π) electrons on its orbital are responsible for the exceptionally high electrical conductivity [51]. Furthermore, the electrical behaviour of graphene can be improved through functionalization [52] and heteroatom doping [53]. Torabi *et al.* [54] synthesised nanocomposite electrodes constituting porous graphene nanoribbons (PGNRs) and carbon black (CB). This PGNRs/CB electrode has a large specific area (1062.5 m^2/g) and capacity of 223.0 F/g at 1.0 A/g current density. Supercapacitor electrodes were developed by intercalating copolymer Pluronic F127 between the layers of reduced graphene oxide (rGO) sheets. The intercalation of copolymer increases the surface area and pore-volume that results in the enhancement of surface wettability and improves its electrochemical performance [55]. In another study, hydrazine reduced graphene hydrogel (GH-Hz) electrode possesses high electrical conductivity, with a specific capacity of 220 F/g at 1 A/g and a high retention capability (around 74%) at a high current density (100 A/g) [56]. Xu *et al.* reported an efficient way to prepare holey graphene oxide through a scalable defect-etching strategy that creates numerous nanopores across the GO plane. Further reduction of holey graphene oxide using H_2O results in three-dimensional hierarchical porous holey graphene hydrogel with significantly enhanced ion transport and surface area [57].

Supplementary Note 2: Data source

Table S1. Information on the literature sources analysed to implement the dataset.

S. No.	Year	Title	Figure /Table	Reference
1	2010	Microstructure and electrochemical double-layer capacitance of carbon electrodes prepared by zinc chloride activation of sugar cane bagasse	Figure 7a	https://doi.org/10.1016/j.jpowsour.2009.08.048
2	2011	Preparation of Highly Conductive Graphene Hydrogels for Fabricating Supercapacitors with High Rate Capability	Figure 5a & 5b	https://doi.org/10.1021/jp204036a
3	2011	Hierarchical porous carbon obtained from animal bone and evaluation in electric double-layer capacitors	Figure 5	https://doi.org/10.1016/j.carbon.2010.10.025
4	2011	Preparation of capacitor's electrode from sunflower seed shell	Figure 2a & 2b	https://doi.org/10.1016/j.biortech.2010.08.110
5	2012	Activated carbons from KOH-activation of argan (Argania spinosa) seed shells as supercapacitor electrodes	Figure 6	doi:10.1016/j.biortech.2012.02.010
6	2012	Preparation of activated carbon from cotton stalk	Figure 6	https://doi.org/10.1007/s10008-012-1946-6
7	2012	Carbonized Chicken Eggshell Membranes with 3D Architectures as High-Performance Electrode Materials for Supercapacitors	Figure 3d	https://doi.org/10.1002/aenm.201100548
8	2013	High-Performance Asymmetric Supercapacitor Based on Nanoarchitectured Polyaniline/Graphene/Carbon Nanotube and Activated Graphene Electrodes	Figure 4d	https://doi.org/10.1021/am4028235
9	2013	Rice husk-derived porous carbons with high capacitance by ZnCl2 activation for supercapacitors	Figure 5b	http://dx.doi.org/10.1016/j.electacta.2013.05.050
10	2013	From coconut shell to porous graphene-like nanosheets for high-power supercapacitors†	Figure 7c	https://doi.org/10.1039/C3TA10897J
11	2013	Tunable N-doped or dual N, S-doped activated hydrothermal carbons derived from human hair and glucose for supercapacitor applications	Figure 4d	https://doi.org/10.1016/j.electacta.2013.06.065
12	2013	Human hair-derived carbon flakes for electrochemical supercapacitors	Figure 5d	https://doi.org/10.1039/C3EE43111H
13	2013	Preparation of activated carbon hollow fibers from ramie at low	Figure 3c	https://doi.org/10.1016/j.biortech.2013.09.026
14	2013	Efficient preparation of biomass-based mesoporous carbons for supercapacitors with both high energy density and high power density	Figure 3a	https://doi.org/10.1016/j.jpowsour.2013.03.174
15	2013	Nitrogen-Doped Porous Graphitic Carbon as an Excellent Electrode Material for Advanced Supercapacitors	Figure 8a	https://doi.org/10.1002/chem.201303345
16	2014	Hierarchical porous and N-doped carbon nanotubes derived from polyaniline for electrode materials in supercapacitors	Figure 6d	https://doi.org/10.1039/C4TA01465K

17	2014	Freestanding 3D mesoporous graphene oxide for high performance energy storage applications	Figure 5c	https://doi.org/10.1039/C4RA08519A
18	2014	Colossal pseudocapacitance in a high functionality–high surface area carbon anode doubles the energy of an asymmetric supercapacitor	Figure 3d	https://doi.org/10.1039/C3EE43979H
19	2014	Importance of open, heteroatom-decorated edges in chemically doped-graphene for supercapacitor applications	Figure 8a	https://doi.org/10.1039/C4TA00936C
20	2014	Shape-controlled porous nanocarbons for high performance supercapacitors	Figure 6d	https://doi.org/10.1039/C3TA15245F
21	2014	A novel route for preparation of high-performance porous carbons from hydrochars by KOH activation	Table 2	https://doi.org/10.1016/j.colsurfa.2014.01.013
22	2014	A high-performance carbon derived from corn stover via microwave and slow pyrolysis for supercapacitors	Table 1	https://doi.org/10.1016/j.jaap.2014.07.010
23	2014	Surfactant-modified chemically reduced graphene	Figure 9b	https://doi.org/10.1039/C4RA03826F
24	2014	Oriented and Interlinked Porous Carbon Nanosheets with an	Figure 4c	https://doi.org/10.1021/cm503845q
25	2014	Superior capacitive performance of active carbons deSuperior capacitive performance of active carbons derived from Enteromorpha prolifera		http://dx.doi.org/10.1016/j.electacta.2014.04.101
26	2014	Hierarchical nitrogen-doped porous carbon with high surface area derived from endothelium corneum gigeriae galli for high-performance supercapacitor	Figure 5d	https://doi.org/10.1016/j.electacta.2014.03.015
27	2014	Direct Synthesis of Highly Porous Interconnected Carbon Nanosheets and Their Application as High- Performance Supercapacitors	Figure 5b	https://doi.org/10.1021/nn501124h
28	2015	Converting biowaste corncob residue into high value added porous carbon for supercapacitor electrodes		https://doi.org/10.1016/j.biortech.2015.04.005
29	2015	Nitrogen-doped hierarchical porous carbon materials prepared from meta-aminophenol formaldehyde resin for supercapacitor with high rate performance	Figure 5/Table 1 & 2	http://dx.doi.org/10.1016/j.electacta.2014.11.075
30	2015	Nitrogen-doped porous carbon derived from biomass waste for high-performance supercapacitor	Figure 2e	http://dx.doi.org/10.1016/j.biortech.2015.07.100
31	2015	Promising biomass-based activated carbons derived from willow catkins for high performance supercapacitors	Figure 7d	http://dx.doi.org/10.1016/j.electacta.2015.03.048
32	2015	Promising Nitrogen-Rich Porous Carbons Derived from One-Step Calcium Chloride Activation of Biomass-Based Waste for High Performance Supercapacitors	Figure 5g & s11	https://doi.org/10.1021/acssuschemeng.5b00926
33	2015	High capacitive performance of exfoliated biochar nanosheets from biomass waste corn cob	Figure 6d	https://doi.org/10.1021/acssuschemeng.5b00926
34	2015	Ultrahigh Surface Area Three-Dimensional Porous Graphitic Carbon	Figure 6c	https://doi.org/10.1021/acscentsci.5b00149

		from Conjugated Polymeric Molecular Framework		
35	2015	Ultrahigh volumetric capacitance and cyclic stability of fluorine and nitrogen co-doped carbon microspheres.		https://doi.org/10.1038/ncomms9503
36	2015	Activated porous carbon prepared from paulownia flower for high performance supercapacitor electrodes	Figure 3d	http://dx.doi.org/10.1016/j.electacta.2014.12.169
37	2015	Hierarchically porous carbon by activation of shiitake mushroom for capacitive energy storage	Figure 5c	http://dx.doi.org/10.1016/j.carbon.2015.05.056
38	2015	Large scale production of biomass-derived nitrogen-doped porous carbon materials for supercapacitors	Figure 6b	http://dx.doi.org/10.1016/j.electacta.2015.04.082
39	2015	Impregnation assisted synthesis of 3D nitrogen-doped porous carbon with high capacitance	Figure 7d	https://doi.org/10.1016/j.carbon.2015.07.058
40	2015	High performance electrode materials for electric double-layer capacitors based on biomass-derived activated carbons	Figure 5g	https://doi.org/10.1016/j.electacta.2015.05.080
41	2015	Solution Processable Holey Graphene Oxide and Its Derived Macrostructures for High-Performance Supercapacitors	Figure 3d, 4h, 5b, 6c, & 6f	https://doi.org/10.1021/acs.nanolett.5b01212
42	2015	Facile self-templating large scale preparation of biomass-derived 3D hierarchical porous carbon for advanced supercapacitors	Figure 5e	https://doi.org/10.1039/C5TA04721H
43	2015	Impregnation assisted synthesis of 3D nitrogen-doped porous carbon with high capacitance	Figure 7d	https://doi.org/10.1016/j.carbon.2015.07.058
44	2015	Electrochemical properties of carbon from oil palm kernel shell for high performance supercapacitors	Figure 8d	https://doi.org/10.1016/j.electacta.2015.05.163
45	2015	Nitrogen, oxygen and phosphorus decorated porous carbons derived from shrimp shells for supercapacitors	Figure 5c	https://doi.org/10.1016/j.electacta.2015.07.094
49	2016	Construction of nitrogen-doped porous carbon buildings using interconnected ultra-small carbon nanosheets for ultra-high rate supercapacitors	Figure 4d	https://doi.org/10.1039/C6TA02570F
50	2016	Renewable Graphene-Like Nitrogen-Doped Carbon Nanosheets as Supercapacitor Electrodes with Integrated High Energy-Power Property	Figure 3c	https://doi.org/10.1039/C6TA02828D
51	2016	A melamine-assisted chemical blowing synthesis of N-doped activated carbon sheets for supercapacitor application (Activated carbon)	Figure 5c	http://dx.doi.org/10.1016/j.jpowsour.2016.04.069
52	2016	Hierarchically Porous N-Doped Carbon Nanosheets Derived From Grapefruit Peels for High-Performance Supercapacitors	Figure 5c	http://dx.doi.org/10.1002/slct.201600133
53	2016	Facile Synthesis of Three-Dimensional Heteroatom-Doped and Hierarchical Egg-Box-Like Carbons Derived from Moringa oleifera Branches for High-Performance Supercapacitors	Figure 5e	https://doi.org/10.1021/acsami.6b10893
54	2016	A shiitake-derived nitrogen/oxygen/phosphorus co-doped	Figure 4c	https://doi.org/10.1039/C6RA13689C

		carbon framework with hierarchical trimodal porosity for high-performance electrochemical capacitors		
55	2016	A Two-Step Etching Route to Ultrathin Carbon Nanosheets for High Performance Electrical Double Layer Capacitors	Figure 5c	https://doi.org/10.1039/C6NR02155G
56	2016	Heteroatom-Doped Porous Carbon Nanosheets: General Preparation and Enhanced Capacitive Properties	Figure 5c	https://doi.org/10.1002/chem.201602922
57	2016	Effect of pristine graphene incorporation on charge storage mechanism of three-dimensional graphene oxide: superior energy and power density retention	Figure 5b	10.1038/srep31555
58	2016	KOH-Activated Porous Carbons Derived from Chestnut Shell with Superior Capacitive Performance	Figure 7d	https://doi.org/10.1002/cjoc.201600320
59	2016	Multi-heteroatom self-doped porous carbon derived from swim bladders for large capacitance supercapacitors	Figure 7c	https://doi.org/10.1039/C6TA06337C
60	2016	Promising porous carbons derived from lotus seedpods with outstanding supercapacitance performance	Table 1/Figure 4b	http://dx.doi.org/10.1016/j.electacta.2016.05.020
61	2016	Preparation and application of capacitive carbon from bamboo shells by one step molten carbonates carbonization	Figure 6c	http://dx.doi.org/10.1016/j.ijhydene.2016.05.083
62	2016	Biomass-Swelling Assisted Synthesis of Hierarchical Porous Carbon Fibers for Supercapacitor Electrodes	Figure 4c & 5c	https://doi.org/10.1021/acsami.5b11558
63	2016	Hierarchical structured carbon derived from bagasse wastes: A simple and efficient synthesis route and its improved electrochemical	Figure 5e	https://doi.org/10.1016/j.jpowsour.2015.10.063
64	2016	Hierarchical Porous Carbon Microtubes Derived from Willow Catkins for Supercapacitor Application	Figure 6e	https://doi.org/10.1039/C5TA09043A
65	2016	Nitrogen-doped interconnected carbon nanosheets from pomelo mesocarps for high performance supercapacitors	Figure 5d	http://dx.doi.org/10.1016/j.electacta.2015.12.195
66	2016	Nitrogen-doped mesoporous carbons for high performance supercapacitors	Table 4	http://dx.doi.org/10.1016/j.apsusc.2016.04.064
67	2016	Pumpkin-Derived Porous Carbon for Supercapacitors with High Performance	Figure 4e	https://doi.org/10.1002/asia.201600303
68	2016	Popcorn-Derived Porous Carbon for Energy Storage and CO2 Capture	Figure 3b	https://doi.org/10.1021/acs.langmuir.6b01953
69	2016	A new route for the fabrication of corn starch-based porous carbon as electrochemical supercapacitor electrode material		https://doi.org/10.1016/j.colsurfa.2016.05.049
70	2016	Preparation of activated carbon from willow leaves and evaluation in electric double-layer- capacitors	Figure 3c	https://doi.org/10.3390/molecules25184255
71	2016	Microporous carbon from a biological waste-stiff silkworm for capacitive energy storage		https://doi.org/10.1016/j.electacta.2016.10.120
72	2017	Extremely high-rate aqueous supercapacitor fabricated using doped carbon nanoflakes with large surface	Figure 3d	https://doi.org/10.1007/s12274-017-1486-6

		area and mesopores at near-commercial mass loading		
73	2017	High performance aqueous supercapacitor based on highly nitrogen doped carbon nanospheres with unimodal mesoporosity .	Figure 4e & 5d	http://dx.doi.org/10.1016/j.jpowsour.2016.10.086
74	2017	Designed formation of hollow particle-based nitrogen-doped carbon nanofibers for high-performance supercapacitors	Figure 5c	https://doi.org/10.1039/C7EE00488E
75	2017	Highly Doped Carbon Nanobelts with Ultrahigh Nitrogen Content as High-Performance Supercapacitor Materials	Figure 4c	https://doi.org/10.1002/smll.201700834
76	2017	Multiscale Pore Network Boosts Capacitance of Carbon Electrodes for Ultrafast Charging	Figure 3c	https://doi.org/10.1021/acs.nanolett.7b00533
77	2017	Porous carbon derived from ailanthus altissima with unique honeycomb-like microstructure for high-performance	Figure 4e	https://doi.org/10.1039/C7NJ01127J
78	2017	Preparation of highly porous carbon through activation of NH4Cl induced hydrothermal microsphere derivation of glucose	Figure 6c	https://doi.org/10.1039/C6RA26141H
79	2017	Hierarchical Hybrids Integrated by Dual Polypyrrole-Based Porous Carbons for Enhanced Capacitive Performance	Figure 5d	https://doi.org/10.1002/chem.201702544
80	2017	Supercapacitor electrode materials with hierarchically structured pores from carbonization of MWCNTs	Table S3	https://doi.org/10.1039/C6NR08987A
81	2017	Engineered Fabrication of Hierarchical Frameworks with Tuned Pore Structure and N,O-Co-Doping for High-Performance Supercapacitors	Figure 3c	https://doi.org/10.1021/acsami.7b09801
82	2017	Enzymatic hydrolysis lignin derived hierarchical porous carbon for supercapacitors in ionic liquids with high power and energy densities	Figure 3c	https://doi.org/10.1039/C7GC00506G
83	2017	Hierarchical nitrogen-doped porous carbon derived from lecithin for high-performance supercapacitors	Figure 5f	https://doi.org/10.1039/C7RA07984B
84	2017	Superior supercapacitive performance of hollow activated carbon nanomesh with hierarchical structure derived from poplar catkins	Figure 7e	http://dx.doi.org/10.1016/j.jpowsour.2017.07.021
85	2017	Biomass based nitrogen-doped structure-tunable versatile porous carbon material	Figure S9a	https://doi.org/10.1039/C7TA02113E
86	2017	Template-free synthesis of N-doped carbon with pillared-layered pores as bifunctional materials for supercapacitor and environmental applications	Figure 5c	http://dx.doi.org/10.1016/j.carbon.2017.03.027
87	2017	Promising nitrogen-doped porous nanosheets carbon for supercapacitors	Figure 6d	https://doi.org/10.1007/s11581-016-1897-5
88	2017	Electrochemical Studies on Corncob Derived Activated Porous Carbon for Supercapacitors Application in Aqueous and Non-aqueous Electrolytes	Figure 6	https://doi.org/10.1016/j.electacta.2017.01.095
89	2017	Preparation of high performance supercapacitor materials by fast pyrolysis of corn gluten meal waste	Figure 4c & 5h	https://doi.org/10.1039/C7SE00029D

90	2017	Enhanced electrochemical performance of straw-based porous carbon fibers for supercapacitor	Figure 6c	https://doi.org/10.1007/s10008-017-3689-x
91	2017	Porous 3D Few-Layer Graphene-like Carbon for Ultrahigh-Power Supercapacitors with Well-Defined Structure–Performance Relationship	Figure 3d & 5d	https://doi.org/10.1002/adma.201604569
92	2017	Fish gill-derived activated carbon for supercapacitor application	Figure 8d	https://doi.org/10.1016/j.jallcom.2016.10.013
93	2017	An activated carbon derived from tobacco waste for use as a supercapacitor electrode material	Figure 7d	https://doi.org/10.1016/S1872-5805(17)60140-9
94	2017	Flute type micropores activated carbon from cotton stalk for high performance supercapacitors	Figure 7a	10.1016/j.jpowsour.2017.05.054
95	2017	N-doped porous reduced graphene oxide as an efficient electrode material for high performance flexible solid-state supercapacitor	Figure 5e	https://doi.org/10.1016/j.apmt.2016.10.002
96	2018	One-pot synthesis of nitrogen-doped ordered mesoporous carbon spheres for high-rate and long-cycle life supercapacitors	Figure 7c	https://doi.org/10.1016/j.carbon.2017.10.084
97	2018	High surface area carbon materials derived from corn stalk core as electrode for supercapacitor		https://doi.org/10.1016/j.diamond.2018.06.018
98	2018	Three-dimensional porous activated carbon derived from loofah sponge biomass for supercapacitor applications	Figure 8e	https://doi.org/10.1016/j.apsusc.2017.11.249
99	2018	Activated biomass carbon made from bamboo as electrode material for supercapacitors	Figure 5c	https://doi.org/10.1016/j.materresbull.2018.03.006
100	2018	Supercapacitor Electrode Based on Activated Carbon Wool Felt	Figure 9b	https://doi.org/10.3390/c4020024
101	2018	Waste Biomass Based-Activated Carbons Derived from Soybean Pods as Electrode Materials for High-Performance Supercapacitors	Figure 4c	https://doi.org/10.1002/slct.201800609
102	2018	A high performance nitrogen-doped porous activated carbon for supercapacitor derived from pueraria	Figure 6c	https://doi.org/10.1016/j.jallcom.2018.02.078
103	2018	Activated carbons from agricultural waste solvothermally doped with sulphur as electrodes for supercapacitors	Figure 5b	https://doi.org/10.1016/j.cej.2017.11.141
104	2018	Hierarchical porous carbon prepared from biomass through a facile method for supercapacitor applications	Figure 5b	https://doi.org/10.1016/j.jcis.2018.06.076
105	2018	N-enriched multilayered porous carbon derived from natural casings for high-performance supercapacitors	Figure 5d	https://doi.org/10.1016/j.apsusc.2018.03.100
106	2018	Activated carbon derived from harmful aquatic plant for high stable supercapacitors	Figure 3g	https://doi.org/10.1016/j.cplett.2017.11.031
107	2018	High performance porous graphene nanoribbons electrodes synthesized via hydrogen plasma and modified by Pt-Ru nanoclusters for charge storage and methanol oxidation	Figure 3d	https://doi.org/10.1016/j.electacta.2018.09.082

108	2018	Sustainable activated carbons from dead ginkgo leaves for supercapacitor electrode active materials	Figure 5f	https://doi.org/10.1016/j.ces.2018.02.004
109	2018	Tailoring Biomass-Derived Carbon for High-Performance	Figure 5c	https://doi.org/10.1039/C7TA09608A
110	2019	Sakura-based activated carbon preparation and its performance in supercapacitor applications	Figure 7g	https://doi.org/10.1039/C8RA09685F
111	2019	Enhancement of the electrochemical properties of commercial coconut shell-based activated carbon by H2O dielectric barrier discharge plasma	Figure 8d	http://dx.doi.org/10.1098/rsos.180872
112	2019	Highly Porous Willow Wood-Derived Activated Carbon for High-Performance Supercapacitor Electrodes	Figure 6c	https://doi.org/10.1021/acsomega.9b01977
113	2019	Oxygen- and Nitrogen-Enriched Honeycomb-Like Porous Carbon from Laminaria japonica with Excellent Supercapacitor Performance in Aqueous Solution	Figure 8a	https://doi.org/10.1021/acssuschemeng.9b01448
114	2019	A sustainable approach to produce activated carbons from pecan nutshell waste for environmentally friendly supercapacitors	Figure 7c	https://doi.org/10.1016/j.carbon.2019.04.017
115	2019	N, S co-doped porous carbons from natural Juncus effuses for high performance supercapacitors	Figure 3e	https://doi.org/10.1016/j.diamond.2019.107577
116	2019	Robust hierarchically interconnected porous carbons derived from discarded Rhus typhina fruits for ultrahigh capacitive performance supercapacitors	Figure 5b	https://doi.org/10.1016/j.jpowsour.2018.12.064
117	2019	Nitrogen self-doped porous carbon with layered structure derived from porcine bladders for high-performance supercapacitors	Figure 5c	https://doi.org/10.1016/j.jcis.2019.02.024
118	2019	Nitrogen-doped microporous carbon derived from a biomass waste metasequoia cone for electrochemical capacitors	Figure 5c	https://doi.org/10.1016/j.jallcom.2019.04.237
119	2019	Low-cost, high-performance supercapacitor based on activated carbon Activated carbon derived from pitaya peel for supercapacitor applications with high capacitance performance	Figure 5a	https://doi.org/10.1016/j.jcis.2018.11.103
120	2020	Activated carbon derived from pitaya peel for supercapacitor applications with high capacitance performance		https://doi.org/10.1016/j.matlet.2020.127339
121	2020	S-doped activated mesoporous carbon derived from the Borassus flabellifer flower as active electrodes for supercapacitors	Table S3	https://doi.org/10.1016/j.matchemphys.2019.122151
122	2020	Advanced porous hierarchical activated carbon derived from agricultural wastes toward high performance supercapacitors	Figure 5e	https://doi.org/10.1016/j.jallcom.2019.153111
123	2020	Activated carbons prepared by indirect and direct CO2 activation of lignocellulosic biomass for supercapacitor electrodes	Figure 8a & 8b	https://doi.org/10.1016/j.renene.2020.03.111

124	2020	A new method of synthesizing hemicellulose-derived porous activated lignocellulosic biomass for supercapacitor electrodes carbon for high-performance supercapacitors		https://doi.org/10.1016/j.micromeso.2019.109707
125	2020	The performance of sulphur doped activated carbon supercapacitors prepared from waste tea		https://doi.org/10.1080/09593330.2019.1575480
126	2020	The use of activated carbon from coffee endocarp for the manufacture of supercapacitors		https://doi.org/10.1007/s10854-020-03123-1
127	2020	Microporous carbon from malva nut for supercapacitors: Effects of primary carbonizations on structures and performances	Figure 5e	https://doi.org/10.1016/j.diamond.2020.107816
128	2020	Seaweed-derived KOH activated biocarbon for electrocatalytic oxygen reduction and supercapacitor applications	Figure 7b	https://doi.org/10.1007/s10934-020-00871-7
129	2020	Soybean-waste-derived activated porous carbons for electrochemical double-layer supercapacitors: Effects of processing parameters	Figure 6e	https://doi.org/10.1016/j.est.2019.101070
130	2020	Boosting the supercapacitor performances of activated carbon with carbon nanomaterials	Figure 4j	https://doi.org/10.1016/j.jpowsour.2019.227678
131	2020	Nano-porous carbon materials derived from different biomasses for high performance supercapacitors	Figure 7	https://doi.org/10.1016/j.ceramint.2019.11.031
132	2020	An ultrasonic-assisted synthesis of rice-straw based porous carbon with high performance symmetric supercapacitors	Figure 6d	https://doi.org/10.1039/C9RA08537H
133	2020	Hydrangea-like N/O codoped porous carbons for high-energy supercapacitors	Figure 6f	https://doi.org/10.1016/j.cej.2020.124208
134	2020	Low-cost and advanced symmetry supercapacitors based on three-dimensional tea waste of porous carbon nanosheets	Figure 5a	https://doi.org/10.1080/10667857.2020.1714902
135	2020	Areca nut–derived porous carbons for supercapacitor and CO2 capture applications	Figure 8	https://doi.org/10.1007/s11581-019-03261-5
136	2020	Hierarchical porous carbon electrode materials for supercapacitor developed from wheat straw cellulosic foam	Figure 5e	https://doi.org/10.1016/j.renene.2019.11.150
137	2020	Heteroatoms-doped hierarchical porous carbon derived from chitin for flexible all-solid-state symmetric supercapacitors	Figure 5d	https://doi.org/10.1016/j.cej.2019.123263
138	2020	Walnut shell-derived hierarchical porous carbon with high performances for electrocatalytic hydrogen evolution and symmetry supercapacitors	Figure 4e	https://doi.org/10.1016/j.ijhydene.2019.10.159
139	2020	Facile preparation of N-O codoped hierarchically porous carbon from alginate particles for high performance supercapacitor	Figure 6e	https://doi.org/10.1016/j.jcis.2019.12.027
140	2020	O/N-co-doped hierarchically porous carbon from carboxymethyl cellulose ammonium for high performance supercapacitors	Figure 6e	https://doi.org/10.1007/s10853-020-04515-8

141	2019	Hierarchical porous carbon microrods derived from albizia flowers for high performance supercapacitors	Figure 6e	https://doi.org/10.1016/j.carbon.2019.02.072
142	2020	Scalable green synthesis of hierarchically porous carbon microspheres by spray pyrolysis for high-performance supercapacitors	Figure 8f	https://doi.org/10.1016/j.cej.2019.122805
143	2020	Hierarchically porous carbon derived from the activation of waste chestnut shells by potassium bicarbonate (KHCO3) for high-performance supercapacitor electrode	Figure 9	https://doi.org/10.1002/er.4970
144	2020	Synthesis of porous carbon nanostructure formation from peel waste for low cost flexible electrode fabrication towards energy storage applications	Table 2	https://doi.org/10.1016/j.est.2020.101735
145	2020	Nitrogen-doped Oxygen-rich Activated Carbon Derived from Longan Shell for Supercapacitors	Figure 7e	10.20964/2020.03.18
146	2020	Mesopore-rich carbon flakes derived from lotus leaves and its ultrahigh performance for supercapacitors	Figure 4f	https://doi.org/10.1016/j.electacta.2019.135481
147	2020	An Ultra-microporous Carbon Material Boosting Integrated Capacitance for Cellulose-Based Supercapacitors	Figure 3C	https ://doi.org/10.1007/s4082 0-020-0393-7)

Supplementary Note 3: Details on the regression models

Ordinary least square (OLS) regression

OLS is one of the most common regression models, where the unknown parameters of linear regression are estimated by lessening the sum of the squares of the differences between the target responses of the sample dataset and the value foreseen by a linear function of explanatory variables [58]. A linear regression can be described as:

$$Y = \beta_0 + \beta_1 X_1 + \beta_2 X_2 + \cdots + \beta_i X_i + \epsilon, \qquad \text{(S1)}$$

where Y is the dependent variable, X_i is the explanatory variable, β_i is the coefficient, and ϵ is the random error term.

Support Vector Regression (SVR) model

SVR is a well-established supervised machine learning approach for predicting discrete values. SVR operates on the same principle as Support Vector Machine (SVM). The primary principle of SVR is to determine the best fit line. An optimal hyperplane defines SVM as a discriminative classifier, whereas – in SVR – the best fit line is the hyperplane with the most point. Support vectors are the results of ideal hyperplanes, which classify unseen datasets that support hyperplanes [59, 60]. The hyperplane in a two-dimensional (2D) region is a line separating into two segments wherein each segment is placed on either side. For instance, multiple line data classification can be done with two distinct datasets (*i.e.,* green and red) and used to propose an affirmative interpretation (see Figure S1). However, selecting an optimal hyperplane is not an easy job, as it should not be noise sensitive, and the generalization of datasets should be accurate [61]. Pertinently, SVM is used to determine the optimized hyperplane that provides considerable minimum distance to the trained dataset. SVR attempts to minimize the difference between the real and predicted values by fitting the best line under a certain threshold value. The distance between the hyperplane and the boundary line is the threshold value (see Figure S1). In mathematical notation, for a 2D space, a line can be used to distinguish linearly separable data. The line can be represented as

$$y = ax + b. \qquad \text{(S2)}$$

By renaming x with x_1 and y with x_2, the equation is modified as

$$ax_1 - x_2 + b = 0. \qquad \text{(S3)}$$

If we substitute $X = (x_1, x_2)$ and $w = (a, -1)$, we get the following:

$$wX + b = 0, \qquad \text{(S4)}$$

which is called the equation of the hyperplane and refers to the SVM.

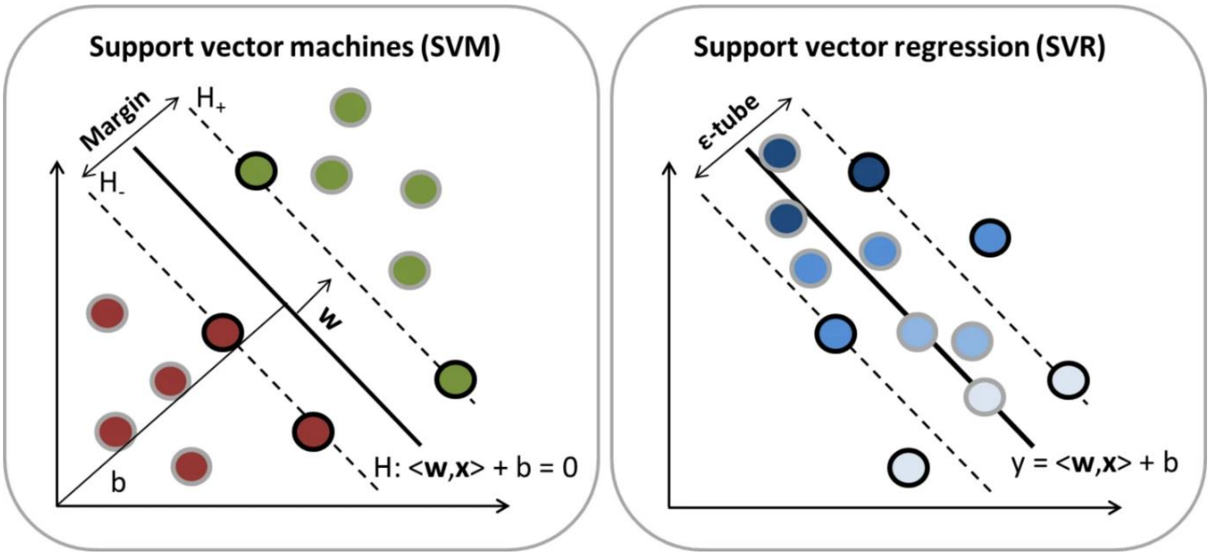

Figure S1: Data classification using (a) SVM and (b) SVR [62].

Consider, the decision boundaries are at any distance say ‘ε’ from the hyperplane. So, these are the lines that we draw at a distance ‘+ε’ and ‘-ε’ from the hyperplane. Then the equations of decision boundary become:

$$wX + b = +\varepsilon, \qquad (S5)$$

$$wX + b = -\varepsilon. \qquad (S6)$$

Thus, any hyperplane that satisfies our SVR should satisfy:

$$-\varepsilon < wX + b < +\varepsilon. \qquad (S7)$$

The key goal here is to choose a decision boundary that has ‘ε’ distance from the initial hyperplane and contains data points closest to the hyperplane.

Decision tree (DT)

DT constructs the regression or classification models based on the data features in the tree’s configuration. In a tree, every node is related to the property of a data feature. Moreover, it either predicts the target value (regression) or predict the target class (classification). The closer the nodes in a tree are, the greater their influence [63]. Some benefits of the DT include:

- It is easy and simple to understand, analyse, and intercept.
- It is capable of handling both categorical and numerical data.

Random forest (RF)

RF is an ensemble learning technique that can perform both regression and classification tasks utilizing the multiple decision trees. During training, the algorithm generates a large number of decision trees using a probabilistic scheme [64]; every tree is trained on a bootstrapped sample of the original training data and finds a randomly selected subset of the input variables to determine a split (for each node). Every tree in the RF makes its own individual prediction or casts a unit vote for the most popular class at input *x*. These predictions are then averaged in case of regression or the majority vote determines the output in case of classification [64]. The core concept is to use numerous decision trees to determine the final output rather than depending on individual decision trees.

Extreme Gradient Boosting (XGBoost) model

XGBoost is one application of gradient boosting machines (GBMs) mainly designed for speed and performance. GBM is the most effective algorithms for supervised learning. In supervised learning, various features in the training data are utilized to predict the target values. XGBoost applies the classification and regression trees (CART) algorithms to a known dataset and categorises the data accordingly [65]. For a dataset consisting of *n* number of samples and *m* number of features, $\mathbb{D} = \{(x_i, y_i)\}(|\mathbb{D}| = n, x_i \epsilon \mathrm{R}^m,\ y_i \epsilon \mathrm{R})$, the expression of an XGBoost algorithm, the total number of CART trees is as follows:

$$\hat{y}_i = \sum_{k=1}^{K} f_k(x_i), f_k \in \mathbf{F}, \tag{S8}$$

where $\mathbf{F} = \{f(x) = w_{q(x)}\}(q\colon \mathrm{R}^m \to T, w \in \mathrm{R}^T)$ is the CART trees space, and $q(x)$ corresponds to an input x to a leaf node of a CART tree. The symbols w and T represent the weight of the node and sum of the leaves in a tree, respectively. As a result, XGBoost calculates a final score by adding up all the weights from each CART tree. The learning goal is to determine the appropriate weights and splitting threshold for each tree node to reduce model complexity. The total loss function of XGBoost is defined as squared loss plus a regularization term:

$$\mathcal{L} = \sum_i l(\hat{y}_i, y_i) + \sum_i \Omega(f_x) = \sum_i (\hat{y}_i - y_i)^2 + \sum_k \left(\gamma T_k + \frac{1}{2}\lambda \|w_k\|^2\right). \tag{S9}$$

By contrast, RFs reduce this loss function by dividing features based on the most significant Gini information gain and by randomly assembling CART trees. XGBoost converts the loss function into a new scoring function that can be used to choose the best threshold [66]:

$$\tilde{\mathcal{L}}^{(t)}(q) = -\frac{1}{2}\sum_{j=1}^{T} \frac{\left(\sum_{i\in I_j} g_i\right)^2}{\sum_{i\in I_j} h_i + \lambda} + \gamma T, \tag{S10}$$

where $\tilde{\mathcal{L}}^{(t)}(q)$ is the second-order approximation of the loss function at the *t*-th iteration, and g_i and h_i are the first and second-order loss gradient on the *i*-th data, respectively. The instance set of a specific leaf node *j* is I_j. As a result, XGBoost can reduce loss iteratively and get better results than other ensemble algorithms.

Supplementary Note 4: Details on the Artificial Neural Network model

For the sake of comprehensiveness, we also developed an artificial neural network (ANN) to predict the capacitance of carbon-based supercapacitors. Several features were selected such as specific surface area (SSA), pore size (PS), pore volume (PV), I_D/I_G ratio, potential window (PW), current density (I), oxygen, nitrogen, and sulphur content in the electrode. Based on the database and the selected features, the ANN was built upon three layers: an input layer, thirteen hidden layer and an output layer. The dataset was divided into training and testing set. For each node of the proposed ANN, the *ReLU* function was applied as the activation function, being the cost function as the mean absolute error. After 250 epochs trainings, the capacitance could be predicted from the designed ANN model. From the regression analysis, the correlation coefficient (R^2) achieved is 0.72, which indicates a fair prediction capability of this model. The other error metrics obtained (*RMSE*, *b'* and *MAPE*) are also indicated in Table S2: overall, ANN shows a slightly lower prediction accuracy as compared to RF and XGBoost models, at least for the considered conditions.

Table S2. Performance analysis of the different ML models.

Model	R^2	RMSE	b'	MAPE
ANN	0.72	47.3	1.06	26.32
RF	0.75	43.96	0.98	27.09
XGBoost	0.79	40.27	0.95	30.08

Supplementary Note 5: Influence of the carbon material percentage on the specific capacitance

We also investigated the impact of carbon material percentage on specific capacitance by refining our database to include the carbon material percentage (C) in the electrode material. Among the 4538 data entries of our clean database, information on carbon material percentage was available for 3117 of them and has been included. We then used an XGBoost model on this refined database to determine the effect of carbon material percentage on specific capacitance. Figure S2 (a) illustrates the correlation between actual and predicted specific capacitance for the dataset considering a 6M KOH electrolyte *viz* R^2 = 0.80, RMSE = 37.09, b' = 0.97, and MAPE = 25.42. Additionally, we performed a feature analysis on the 6M KOH electrolyte dataset, as shown in Figure S2 (b), which revealed that the specific surface area (SSA), heteroatom doping (N%), and pore size (PS) were still the significant factors influencing specific capacitance, coherently with Figure 6 (b).

To further enhance the regression performance, we refined the datasets based on a specific electrolyte (6M KOH) and testing methods (two and three electrode). The effect of this refinement on the data analysis is shown in Figures S2 (c) and (e), where most data points were positioned near the diagonal line, indicating a strong correlation between actual and predicted specific capacitance values. Moreover, the accuracy of the regression was confirmed by the improved statistics of the XGBoost model fitting for both the two-electrode method (R^2 = 0.90, RMSE = 25.26, b' = 0.96, and MAPE = 18.23) and the three-electrode method (R^2 = 0.91, RMSE = 23.25, b' = 1.007, and MAPE = 8.39). We conducted feature analyses on supercapacitors with a 6M KOH electrolyte, as depicted in Figures S2 (d) and (f). Our findings revealed that PS, SSA, and defects were the major contributors to the capacitive performance in the two-electrode testing method, while PV, SSA, and PS were the major contributors in the three-electrode method.

It is important to note that the percentage of carbon weight is a significant factor in determining the specific capacitance; however, since our database only includes carbon-based material, we were unable to distinguish it in our analysis. Consequently, we found that other parameters appear to be more prominent.

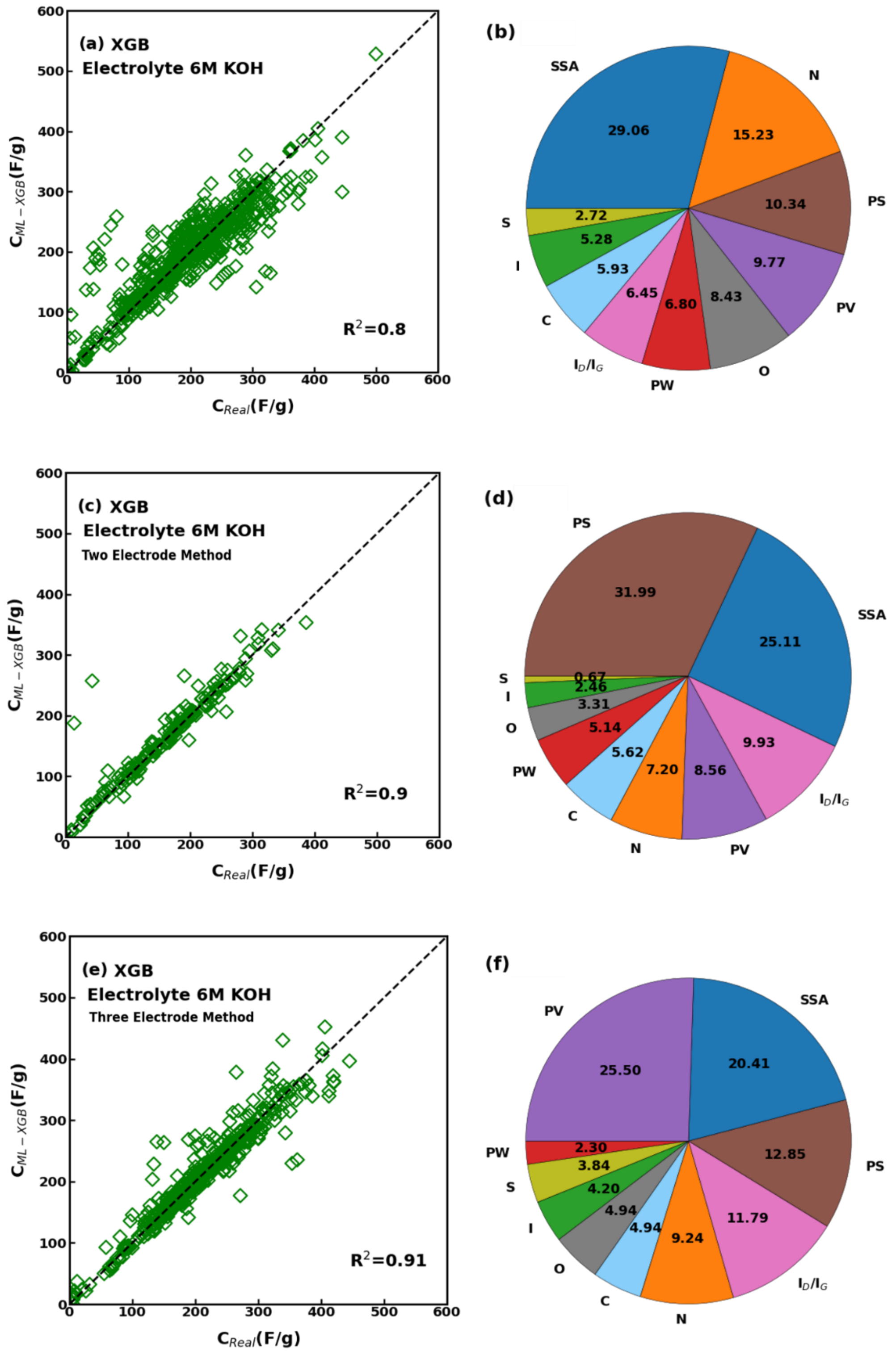

Figure S2: (a) Comparison between the predicted and actual specific capacitance and (b) feature analysis for the subset of data having 6M KOH electrolyte. (c) Comparison between the predicted and actual specific capacitance and (d) feature analysis for the subset of data having 6M KOH electrolyte and measured by two-electrode method. (e) Comparison between the predicted and actual specific capacitance and (f) feature analysis for the subset of data having 6M KOH electrolyte and measured by three-electrode method. These analyses were carried out also considering the carbon material percentage (C) in the electrode.

Supplementary Note 6: Details of the hyperparameters of the ML models in this study

**Hyperparameters**	**DT**	**SVR**	**RF**	**XGBoost**
Gamma	-	10	-	0.3
Max_depth	10	-	16	3
Max_features	Auto	-	-	-
Min_samples_split	5	-	-	-
N_estimators	-	-	1000	500
Min_child_weight	-	-	-	4
C	-	10	-	-
Kernel	-	RBF	-	-
Epsilon	-	0.1	-	-

Table S3. Hyperparameters of the different ML models used in this study for the Figure 4.

**Cases**	Gamma	Max_depth	Min_child_weight	N_estimators
Three Electrode	0.5	3	5	500
Two Electrode	0.4	3	4	500
6M KOH	0.3	3	5	300
1M H_2SO_4	0.3	3	5	200
Three electrode & 6M KOH	0.5	4	4	500
Two electrode & 6M KOH	0.5	4	4	500
AC	0.3	2	4	500
HPC	0.3	2	5	100
HA	0.3	2	4	300

Table S4. Hyperparameters of the XGBoost model used in this study for the Figures 5, 6, 7, and 8 respectively.

References

1. Hu, F., et al., *Engineered fabrication of hierarchical frameworks with tuned pore structure and N, O-co-doping for high-performance supercapacitors.* ACS applied materials & interfaces, 2017. **9**(37): p. 31940-31949.
2. Li, X., et al., *Supercapacitor electrode materials with hierarchically structured pores from carbonization of MWCNTs and ZIF-8 composites.* Nanoscale, 2017. **9**(6): p. 2178-2187.
3. Li, Z., et al., *Hierarchical Hybrids Integrated by Dual Polypyrrole-Based Porous Carbons for Enhanced Capacitive Performance.* Chemistry–A European Journal, 2017. **23**(54): p. 13474-13481.
4. Zhai, Y., et al., *Carbon materials for chemical capacitive energy storage.* Advanced materials, 2011. **23**(42): p. 4828-4850.
5. Kawano, T., et al., *Preparation of activated carbon from petroleum coke by KOH chemical activation for adsorption heat pump.* Applied thermal engineering, 2008. **28**(8-9): p. 865-871.
6. Bi, Z., et al., *Biomass-derived porous carbon materials with different dimensions for supercapacitor electrodes: a review.* Journal of materials chemistry a, 2019. **7**(27): p. 16028-16045.
7. Yang, H., et al., *Biomass-derived porous carbon materials for supercapacitor.* Frontiers in Chemistry, 2019. **7**: p. 274.
8. Tomczyk, A., Z. Sokołowska, and P. Boguta, *Biochar physicochemical properties: pyrolysis temperature and feedstock kind effects.* Reviews in Environmental Science and Bio/Technology, 2020. **19**(1): p. 191-215.
9. Williams, P.T. and A.R. Reed, *Development of activated carbon pore structure via physical and chemical activation of biomass fibre waste.* Biomass and bioenergy, 2006. **30**(2): p. 144-152.
10. Funke, A. and F. Ziegler, *Hydrothermal carbonization of biomass: a summary and discussion of chemical mechanisms for process engineering.* Biofuels, Bioproducts and Biorefining, 2010. **4**(2): p. 160-177.
11. Ma, X., et al., *Activated porous carbon with an ultrahigh surface area derived from waste biomass for acetone adsorption, CO2 capture, and light hydrocarbon separation.* ACS Sustainable Chemistry & Engineering, 2020. **8**(31): p. 11721-11728.
12. Lu, W., et al., *Activated carbon derived from pitaya peel for supercapacitor applications with high capacitance performance.* Materials Letters, 2020. **264**: p. 127339.
13. Qu, W.-H., et al., *Converting biowaste corncob residue into high value added porous carbon for supercapacitor electrodes.* Bioresource technology, 2015. **189**: p. 285-291.
14. Li, J. and Q. Wu, *Water bamboo-derived porous carbons as electrode materials for supercapacitors.* New Journal of Chemistry, 2015. **39**(5): p. 3859-3864.
15. Li, J. and Q. Wu, *Activated carbon derived from harmful aquatic plant for high stable supercapacitors.* Chemical Physics Letters, 2018. **691**: p. 238-242.
16. Bai, S., et al., *Pumpkin-Derived Porous Carbon for Supercapacitors with High Performance.* Chemistry–An Asian Journal, 2016. **11**(12): p. 1828-1836.
17. Lin, H., et al., *A new method of synthesizing hemicellulose-derived porous activated carbon for high-performance supercapacitors.* Microporous and Mesoporous Materials, 2020. **292**: p. 109707.
18. Wang, Y., et al., *Heteroatoms-doped hierarchical porous carbon derived from chitin for flexible all-solid-state symmetric supercapacitors.* Chemical Engineering Journal, 2020. **384**: p. 123263.

19. Abbas, A. and S. Ansumali, *Global potential of rice husk as a renewable feedstock for ethanol biofuel production.* BioEnergy Research, 2010. **3**(4): p. 328-334.
20. Della, V.P., I. Kühn, and D. Hotza, *Characterization of rice husk ash for use as raw material in the manufacture of silica refractory.* Química Nova, 2001. **24**: p. 778-782.
21. Ludueña, L., et al., *Nanocellulose from rice husk following alkaline treatment to remove silica.* BioResources, 2011. **6**(2): p. 1440-1453.
22. Guo, Y., et al., *Performance of electrical double layer capacitors with porous carbons derived from rice husk.* Materials Chemistry and Physics, 2003. **80**(3): p. 704-709.
23. He, X., et al., *Efficient preparation of biomass-based mesoporous carbons for supercapacitors with both high energy density and high power density.* Journal of Power Sources, 2013. **240**: p. 109-113.
24. Zhang, Z., et al., *A novel route for preparation of high-performance porous carbons from hydrochars by KOH activation.* Colloids and Surfaces A: Physicochemical and Engineering Aspects, 2014. **447**: p. 183-187.
25. Chen, H., et al., *High specific surface area rice hull based porous carbon prepared for EDLCs.* International Journal of Electrochemical Science, 2012. **7**(6): p. 4889-4897.
26. Yu, K., et al., *High surface area carbon materials derived from corn stalk core as electrode for supercapacitor.* Diamond and Related Materials, 2018. **88**: p. 18-22.
27. Wang, D., et al., *High performance electrode materials for electric double-layer capacitors based on biomass-derived activated carbons.* Electrochimica Acta, 2015. **173**: p. 377-384.
28. Karnan, M., et al., *Electrochemical studies on corncob derived activated porous carbon for supercapacitors application in aqueous and non-aqueous electrolytes.* Electrochimica Acta, 2017. **228**: p. 586-596.
29. Cheng, B.-H., et al., *Preparation of high performance supercapacitor materials by fast pyrolysis of corn gluten meal waste.* Sustainable Energy & Fuels, 2017. **1**(4): p. 891-898.
30. Jin, H., et al., *A high-performance carbon derived from corn stover via microwave and slow pyrolysis for supercapacitors.* Journal of Analytical and Applied Pyrolysis, 2014. **110**: p. 18-23.
31. Liang, T., et al., *Popcorn-derived porous carbon for energy storage and CO2 capture.* Langmuir, 2016. **32**(32): p. 8042-8049.
32. Rajabathar, J.R., et al., *Synthesis of porous carbon nanostructure formation from peel waste for low cost flexible electrode fabrication towards energy storage applications.* Journal of Energy Storage, 2020. **32**: p. 101735.
33. Ma, F., et al., *Sakura-based activated carbon preparation and its performance in supercapacitor applications.* RSC advances, 2019. **9**(5): p. 2474-2483.
34. Chang, J., et al., *Activated porous carbon prepared from paulownia flower for high performance supercapacitor electrodes.* Electrochimica Acta, 2015. **157**: p. 290-298.
35. Zhang, G., et al., *Activated biomass carbon made from bamboo as electrode material for supercapacitors.* Materials Research Bulletin, 2018. **102**: p. 391-398.
36. Wang, X., et al., *Enhancement of the electrochemical properties of commercial coconut shell-based activated carbon by H2O dielectric barrier discharge plasma.* Royal Society open science, 2019. **6**(2): p. 180872.
37. Phiri, J., et al., *Highly porous willow wood-derived activated carbon for high-performance supercapacitor electrodes.* ACS omega, 2019. **4**(19): p. 18108-18117.

38. Perez-Salcedo, K., et al., *Seaweed-derived KOH activated biocarbon for electrocatalytic oxygen reduction and supercapacitor applications.* Journal of Porous Materials, 2020. **27**(4): p. 959-969.
39. Zhou, X.-L., et al., *Preparation and application of hierarchical porous carbon materials from waste and biomass: A review.* Waste and Biomass Valorization, 2021. **12**(4): p. 1699-1724.
40. Liu, Y., et al., *Biomass-swelling assisted synthesis of hierarchical porous carbon fibers for supercapacitor electrodes.* ACS applied materials & interfaces, 2016. **8**(42): p. 28283-28290.
41. Feng, H., et al., *Hierarchical structured carbon derived from bagasse wastes: A simple and efficient synthesis route and its improved electrochemical properties for high-performance supercapacitors.* Journal of Power Sources, 2016. **302**: p. 164-173.
42. Zhou, X., et al., *Biomass based nitrogen-doped structure-tunable versatile porous carbon materials.* Journal of Materials Chemistry A, 2017. **5**(25): p. 12958-12968.
43. Gou, G., et al., *Hierarchical porous carbon electrode materials for supercapacitor developed from wheat straw cellulosic foam.* Renewable Energy, 2020. **149**: p. 208-216.
44. Zhao, Y., et al., *Facile preparation of NO codoped hierarchically porous carbon from alginate particles for high performance supercapacitor.* Journal of colloid and interface science, 2020. **563**: p. 414-425.
45. Wang, D., et al., *Nitrogen self-doped porous carbon with layered structure derived from porcine bladders for high-performance supercapacitors.* Journal of colloid and interface science, 2019. **542**: p. 400-409.
46. Shaheen Shah, S., et al., *Preparation of Sulfur-doped Carbon for Supercapacitor Applications: A Review.* ChemSusChem, 2022. **15**(1): p. e202101282.
47. Li, Y., et al., *Nitrogen and sulfur co-doped porous carbon nanosheets derived from willow catkin for supercapacitors.* Nano energy, 2016. **19**: p. 165-175.
48. Wan, L., et al., *Heteroatom-doped porous carbons derived from lotus pollen for supercapacitors: Comparison of three activators.* Journal of Alloys and Compounds, 2021. **859**: p. 158390.
49. Demir, M., et al., *Lignin-derived heteroatom-doped porous carbons for supercapacitor and CO2 capture applications.* International journal of energy research, 2018. **42**(8): p. 2686-2700.
50. Razmjooei, F., et al., *Urine to highly porous heteroatom-doped carbons for supercapacitor: A value added journey for human waste.* Scientific reports, 2017. **7**(1): p. 1-14.
51. Fuhrer, M.S., C.N. Lau, and A.H. MacDonald, *Graphene: materially better carbon.* MRS bulletin, 2010. **35**(4): p. 289-295.
52. Radovic, L.R., et al., *Graphene functionalization: Mechanism of carboxyl group formation.* Carbon, 2018. **130**: p. 340-349.
53. Wang, X.-Y., et al., *Bottom-up synthesis of heteroatom-doped chiral graphene nanoribbons.* Journal of the American Chemical Society, 2018. **140**(29): p. 9104-9107.
54. Torabi, M., R.K. Shervedani, and A. Amini, *High performance porous graphene nanoribbons electrodes synthesized via hydrogen plasma and modified by Pt-Ru nanoclusters for charge storage and methanol oxidation.* Electrochimica Acta, 2018. **290**: p. 616-625.
55. Ke, Q., et al., *Surfactant-modified chemically reduced graphene oxide for electrochemical supercapacitors.* RSC advances, 2014. **4**(50): p. 26398-26406.

56. Zhang, L. and G. Shi, *Preparation of highly conductive graphene hydrogels for fabricating supercapacitors with high rate capability*. The Journal of Physical Chemistry C, 2011. **115**(34): p. 17206-17212.
57. Xu, Y., et al., *Solution processable holey graphene oxide and its derived macrostructures for high-performance supercapacitors.* Nano letters, 2015. **15**(7): p. 4605-4610.
58. Łukawska-Matuszewska, K. and J.A. Urbański, *Prediction of near-bottom water salinity in the Baltic Sea using Ordinary Least Squares and Geographically Weighted Regression models.* Estuarine, Coastal and Shelf Science, 2014. **149**: p. 255-263.
59. Aggarwal, C.C. *Data classification*. in *Data mining*. 2015. Springer.
60. Zhang, F. and L.J. O'Donnell, *Support vector regression*, in *Machine learning*. 2020, Elsevier. p. 123-140.
61. Battineni, G., N. Chintalapudi, and F. Amenta, *Machine learning in medicine: Performance calculation of dementia prediction by support vector machines (SVM).* Informatics in Medicine Unlocked, 2019. **16**: p. 100200.
62. Rodríguez-Pérez, R. and J. Bajorath, *Evolution of support vector machine and regression modeling in chemoinformatics and drug discovery.* Journal of Computer-Aided Molecular Design, 2022. **36**(5): p. 355-362.
63. Swain, P.H. and H. Hauska, *The decision tree classifier: Design and potential.* IEEE Transactions on Geoscience Electronics, 1977. **15**(3): p. 142-147.
64. Breiman, L., et al., *Classification and regression trees*. 2017: Routledge.
65. Dhaliwal, S.S., A.-A. Nahid, and R. Abbas, *Effective intrusion detection system using XGBoost.* Information, 2018. **9**(7): p. 149.
66. Chen, T. and C. Guestrin. *Xgboost: A scalable tree boosting system*. in *Proceedings of the 22nd acm sigkdd international conference on knowledge discovery and data mining*. 2016.
